# Functional analysis of a wheat class III peroxidase gene, *TaPer12-3A*, in seed dormancy and germination

**DOI:** 10.1186/s12870-024-05041-4

**Published:** 2024-04-24

**Authors:** Wei Gao, Yating Jiang, Xiaohu Yang, Ting Li, Litian Zhang, Shengnan Yan, Jiajia Cao, Jie Lu, Chuanxi Ma, Cheng Chang, Haiping Zhang

**Affiliations:** College of Agronomy, Anhui Agricultural University, Key Laboratory of Wheat Biology and Genetic Improvement on Southern Yellow & Huai River Valley, Ministry of Agriculture and Rural Affairs, Hefei, Anhui 230036 China

**Keywords:** Wheat, Class III peroxidase, Seed dormancy, Pre-harvest sprouting, Abscisic acid

## Abstract

**Background:**

Class III peroxidases (PODs) perform crucial functions in various developmental processes and responses to biotic and abiotic stresses. However, their roles in wheat seed dormancy (SD) and germination remain elusive.

**Results:**

Here, we identified a wheat class III POD gene, named *TaPer12-3A*, based on transcriptome data and expression analysis. *TaPer12-3A* showed decreasing and increasing expression trends with SD acquisition and release, respectively. It was highly expressed in wheat seeds and localized in the endoplasmic reticulum and cytoplasm. Germination tests were performed using the transgenic *Arabidopsis* and rice lines as well as wheat mutant mutagenized with ethyl methane sulfonate (EMS) in Jing 411 (J411) background. These results indicated that *TaPer12-3A* negatively regulated SD and positively mediated germination. Further studies showed that *TaPer12-3A* maintained H_2_O_2_ homeostasis by scavenging excess H_2_O_2_ and participated in the biosynthesis and catabolism pathways of gibberellic acid and abscisic acid to regulate SD and germination.

**Conclusion:**

These findings not only provide new insights for future functional analysis of *TaPer12-3A* in regulating wheat SD and germination but also provide a target gene for breeding wheat varieties with high pre-harvest sprouting resistance by gene editing technology.

**Supplementary Information:**

The online version contains supplementary material available at 10.1186/s12870-024-05041-4.

## Background

Wheat (*Triticum aestivum* L.) is cultivated across the globe and constitutes over 20% of the human caloric intake. However, rainfall or hot and humid weather before harvest often causes pre-harvest sprouting (PHS) in wheat, which significantly reduces grain yield and quality [1, 2]. Seed dormancy (SD) is the main factor controlling PHS resistance, which is determined by genetic and environmental factors (e.g., temperature, light, and humidity) [3]. Particularly, high temperature helps to break SD during seed development in wheat [4]. If rainfall occurs during harvest, the losses caused by PHS are difficult to estimate, thereby leading to a potential risk for wheat production [5, 6]. To minimize PHS damage, it is necessary to breed wheat varieties with strong SD and PHS resistance. Thus far, only eight genes associated with SD or PHS resistance have been identified through map-based and homology-based cloning techniques, including *TaVp1* [7], *TaMFT* (*TaPHS1*) [8], *TaDOG1L1* [9], *TaMKK3-A* [10], *TaSdr* [11], *TaQsd1* [12], *TaMyb10-D* [13], and *TaPI4K-2A* [14]. Consequently, exploring additional genes associated with SD and understanding their functions and molecular mechanisms will help to breed wheat varieties with high PHS resistance, thereby minimizing PHS damages.

Reactive oxygen species (ROS) are produced by the reduction of oxygen to superoxide (O_2_^−.^), hydrogen peroxide (H_2_O_2_), hydroxyl radical (HO^.^), and singlet oxygen (^1^O_2_), and are byproducts of many metabolic pathways. ROS, particularly H_2_O_2_, play a fundamental signaling role in plant growth and development, as well as in the response to biotic and abiotic stresses [15]. During seed germination, mitochondria produce sufficient ATP to support germination accompanied with ROS (H_2_O_2_) production, indicating that H_2_O_2_ is closely related with SD release and germination [16, 17]. Studies have indicated that H_2_O_2_ affected SD release and germination by mediating the biosynthesis and catabolism of gibberellic acid (GA) and abscisic acid (ABA) [18, 19]. Although H_2_O_2_ accumulation acts as a positive signal in SD release and germination, excessive H_2_O_2_ accumulation can cause oxidative damage and even complete inactivation of intracellular macromolecules, leading to the disturbance of regulation and metabolism in plants [20]. Ishibashi et al. [21] found a direct correlation between H_2_O_2_ scavenging enzyme activity and germinability during the developmental stages of wheat seeds. In wheat seeds, endogenous ascorbic acid content and catalase activity were significantly higher in Shirogane-Komugi with high germinability than in Norin61 with low germinability. These findings demonstrate that H_2_O_2_ scavenging enzymes are related to seed germinability, and excessive H_2_O_2_ scavenging is conducive to rapid seed germination.

Studies showed that class III PODs also played a crucial role in the effective scavenging of ROS (H_2_O_2_) [22–24]. In sacred lotus (*Nelumbo nucifera*), Chen et al. [25] identified a seed-specific peroxiredoxin gene named *NnPER1*. They observed a significant upregulation in the transcription and protein accumulation of NnPER1 during the initial phase of seed imbibition, a process commonly linked to elevated ROS production. The overexpression of *NnPER1* in *Arabidopsis* resulted in an improved seed germination percentage (GP) and longevity of the transgenic plants. Additionally, ROS levels were also lower in transgenic seeds than in wild-type (WT) seeds. These findings confirmed that the ectopic expression of *NnPER1* enhances the seed germinability of transgenic *Arabidopsis* plants by effectively detoxifying ROS. In rice, Wang et al. [26] reported that overexpression and knockout of *PER1A*, which encodes a key player in the detoxification pathway, increased seed germinability and decreased seed vigor of transgenic plants, respectively. Further studies revealed that PER1A interacted with bZIP23 to regulate seed germination by the ABA signaling pathway [26]. These findings suggest that POD plays a vital role in SD release and germination and crosstalk with the ABA signaling pathway. Nevertheless, the function of POD in wheat SD and germination remains unclear.

In this study, a class III POD gene (*TraesCS3A02G510600*, designated *TaPer12-3A*) was identified based on transcriptome data and expression analysis. Subsequently, we performed germination tests using the transgenic *Arabidopsis* and rice lines and wheat EMS mutant, and conducted molecular, physiological, and biochemical analyses to confirm the role of *TaPer12-3A* in SD and germination. We also investigated the subcellular localization of the TaPer12-3A protein. These data are valuable to broaden our understanding of the molecular network of SD and germination and provide a target for breeding wheat varieties resistant to PHS.

## Results

### Identification and sequence analysis of *TaPer12-3A*

High temperature during seed development decreases dormancy levels of wheat seeds [4]. Based on our previous transcriptome data from wheat landrace WTB seeds treated with high temperature [27] and tissue expression analysis [28], the *TraesCS3A02G510600* gene (named *TaPer12-3A*) was highly expressed in wheat seeds and significantly upregulated by high temperature (Fig. S1A and B; Table S2). Combining the decreased dormancy level of WTB seeds and high expression of *TaPer12-3A* after high temperature treatment, we suggest that *TaPer12-3A* plays a role in dormancy release and germination. Thus, we selected this gene for further study.

**Fig. 1:**
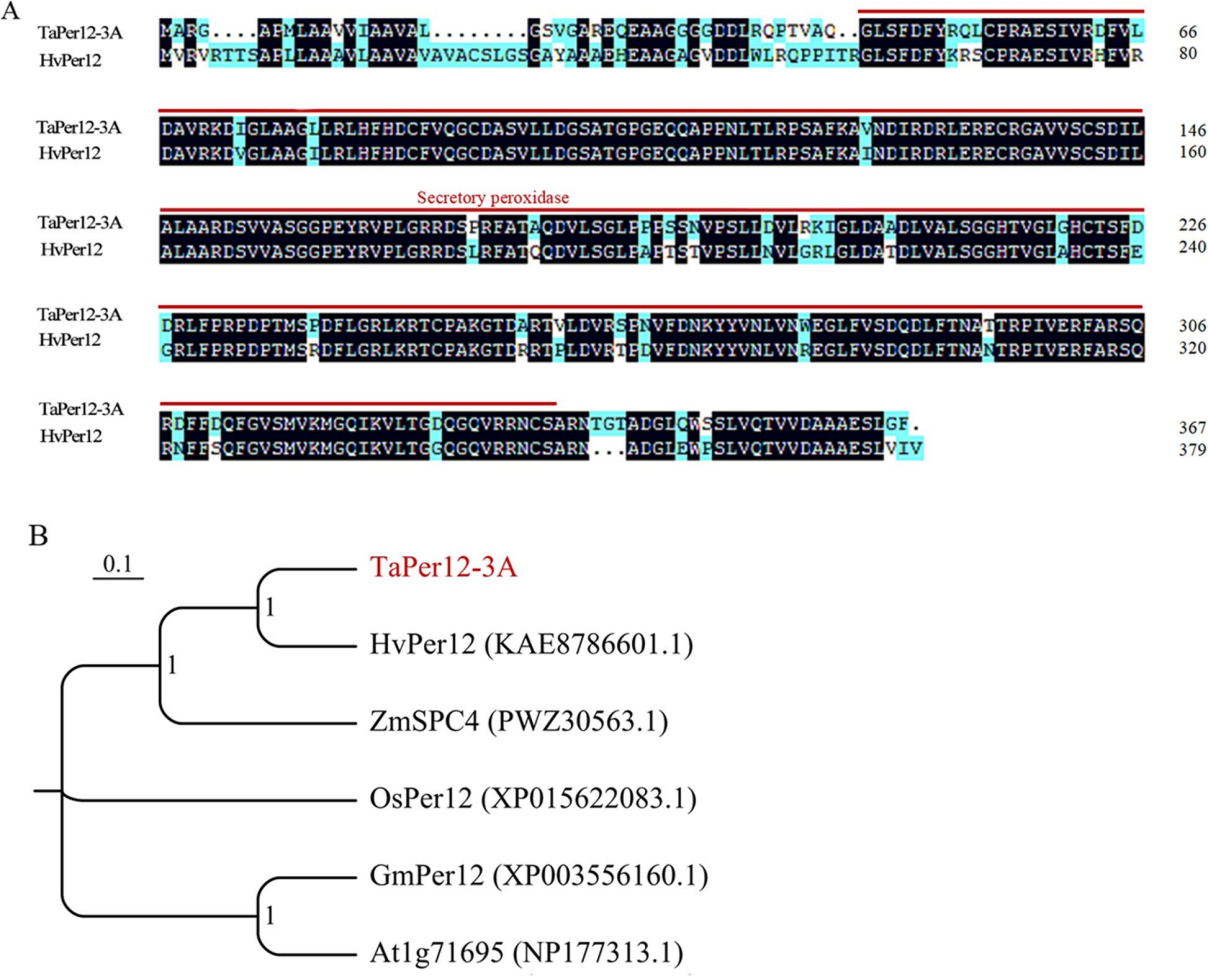
Sequence analysis of the TaPer12-3A protein. **A** Comparative sequence analysis of TaPer12 and HvPer12 amino acids. The red line represents the secretory peroxidase domain. **B** Homologous phylogenetic tree of the TaPer12-3A protein. MEGA v.7.0 was used to produce the neighbor-joining tree with 1000 bootstrap replicates. Initial letters indicate different plants: Ta (*Triticum aestivum*), Hv (*Hordeum vulgare*), Zm (*Zea mays*), Os (*Oryza sativa*), Gm (*Glycine max*), and At (*Arabidopsis thaliana*)

The genomic sequence of *TaPer12-3A* was cloned from WTB, containing a 1414-bp open reading frame encoding 367 amino acids. The phylogenetic tree showed that TaPer12-3A had the closest affinity to HvPer12 (KAE8786601.1) with 80.68% similarity. The TaPer12-3A protein contained the structural domain of secretory POD (CD00693), which is typical of class III POD (Fig. 1A and B).

### Expression analysis of *TaPer12-3A*

**Fig. 2:**
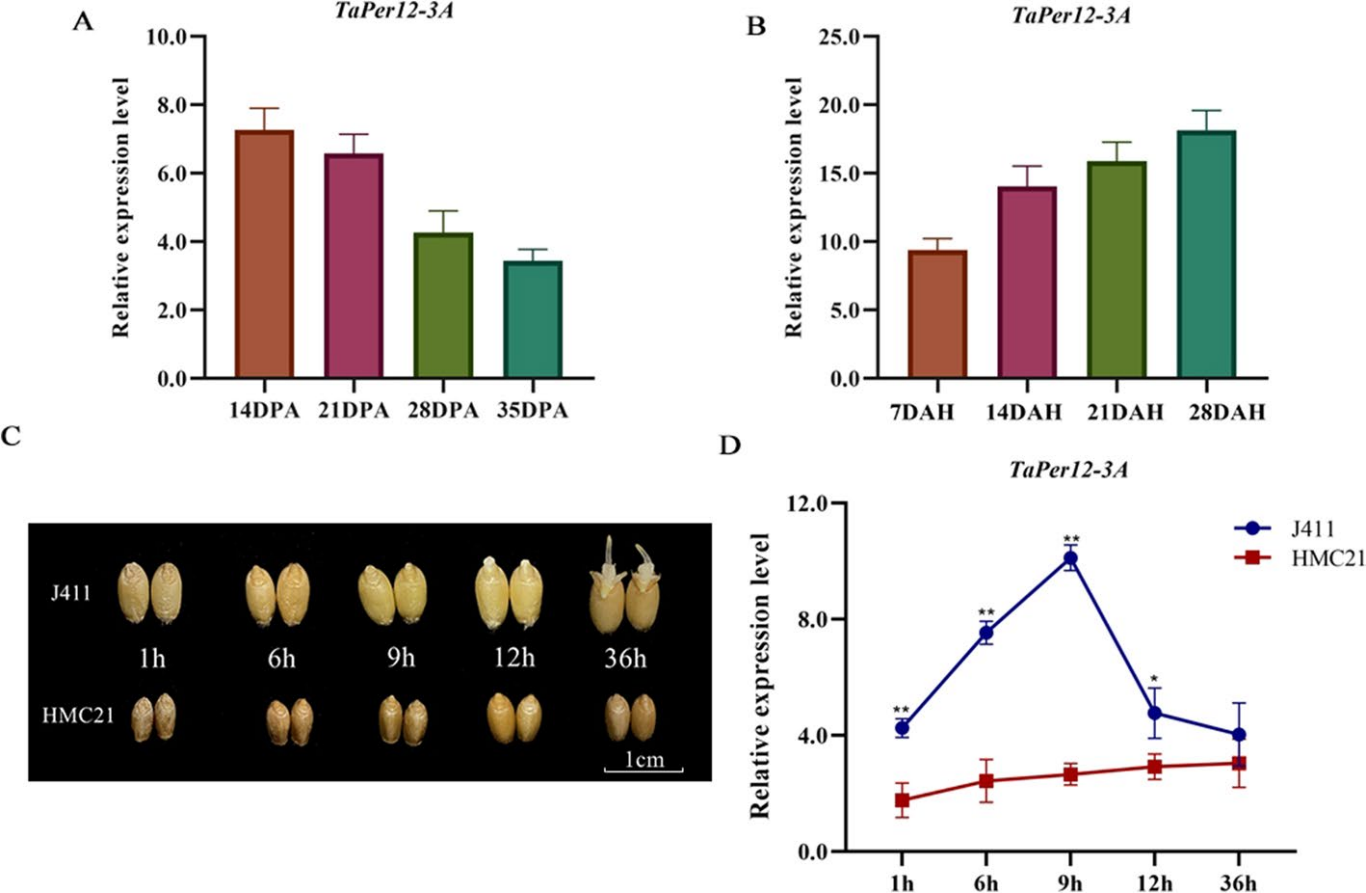
Expression analysis of *TaPer12-3A*. **A** Expression patterns of *TaPer12-3A* at different growth stages (14, 21, 28, and 35 days post-anthesis [DPA]) in the wheat landrace Waitoubai (WTB). **B** Expression patterns of *TaPer12-3A* at different post-ripening stages (7, 14, 21, and 28 days after harvest [DAH]) in WTB. **C** Seed images of Jing 411 (J411) and Hongmangchun 21 (HMC21) imbibed at different time periods. **D** Expression profiles of *TaPer12-3A* at different stages after seed imbibition in J411 and HMC21. *P < 0.05 and **P < 0.01 indicate significance and extreme significance, respectively

We further investigated the expression patterns of *TaPer12-3A* by qRT-PCR and found that the expression level of *TaPer12-3A* was upregulated after high temperature treatment, similar to the transcriptome data (Fig. S1C). In addition, *TaPer12-3A* was highly expressed in seeds compared with roots, stems, leaves, and spikes (Fig. S1D; Table S2). It is well known that seed dormancy is established during seed development and gradually released during post-ripening. Therefore, we further analyzed the association of *TaPer12-3A* with SD and germination at different developmental stages (14, 21, 28, and 35 days post-anthesis [DPA], representing SD acquisition) and post-ripening stages (7, 14, 21, and 28 days after harvest [DAH], representing SD release). The results indicated that the expression level of *TaPer12-3A* decreased during SD acquisition and increased during SD release (Fig. 2A and B). In addition, we investigated the expression patterns of *TaPer12-3A* at several time points after imbibition (1, 6, 9, 12, and 36 h, representing SD release) in the seeds of Hongmangchun 21 (HMC21, strong dormancy) and Jing 411 (J411, weak dormancy) (Fig. 2C). The results showed that *TaPer12-3A* was highly expressed in J411 seeds and low expressed in HMC21 seeds after imbibition (Fig. 2D). Based on these, we suggest that *TaPer12-3A* may promote SD release and germination.

### Role of *TaPer12-3A* in *Arabidopsis* and rice SD and germination

**Fig. 3:**
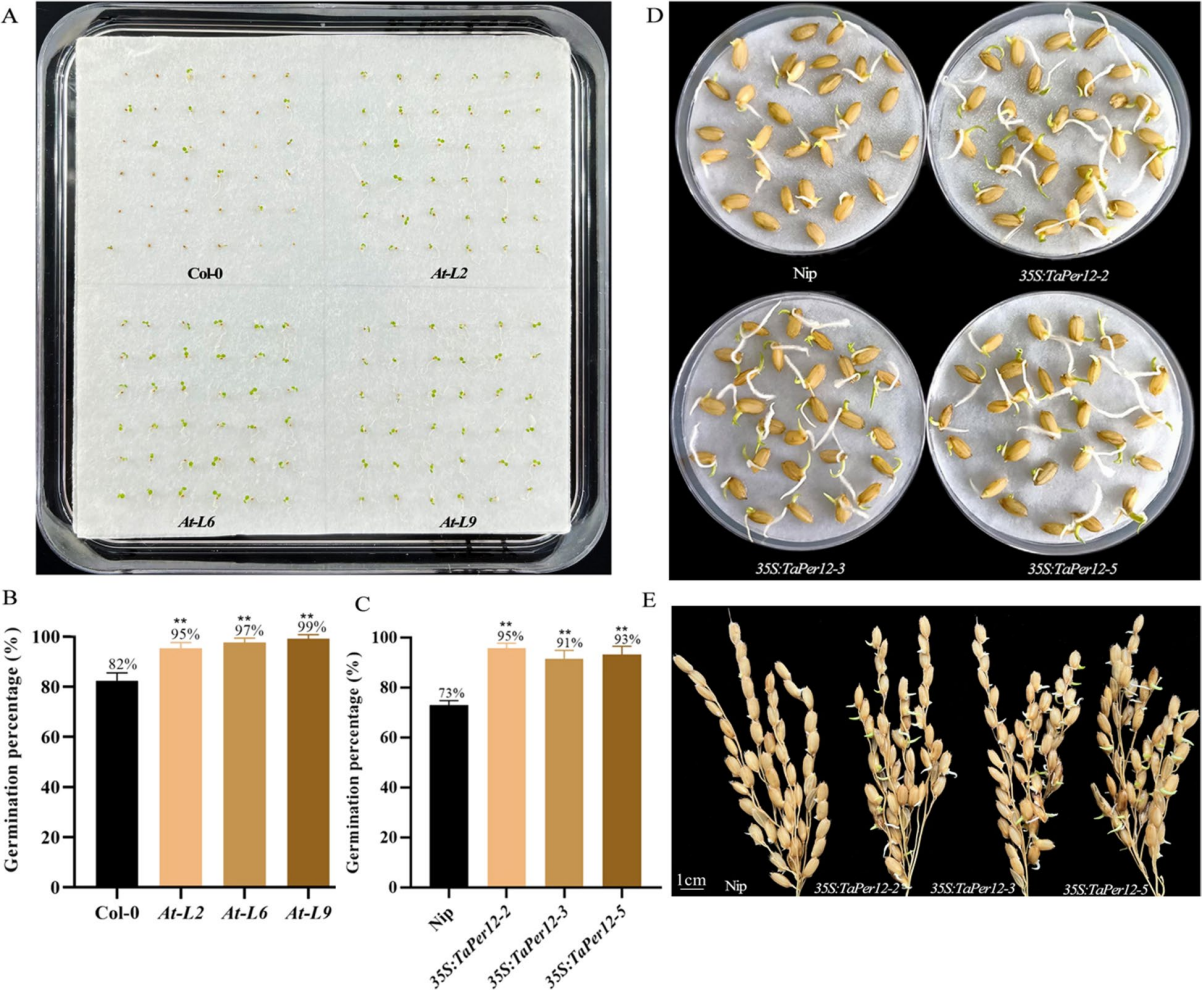
Expression patterns of *TaPer12-3A* in overexpression rice lines and germination tests of transgenic rice seeds. **A** Germination tests of the overexpression *Arabidopsis* (*At-L2/-6/-9*) and Col-0 seeds on the 7th day. **B** Germination percentages of overexpression *Arabidopsis* (*At-L2/-6/-9*) and Col-0 seeds on the 7th day. **P < 0.01 indicates extreme significance. **C** Germination percentages of overexpression rice (*35S:TaPer12*) and Nip seeds on the 7th day. **P < 0.01 indicates extreme significance. **D**, **E** Germination tests of the freshly collected overexpression rice (*35S:TaPer12*) and Nip seeds and panicles. The freshly collected rice seeds and panicles germinated for 7 days

*Arabidopsis* and rice are excellent model plants for functional studies due to the ease of generating transgenic lines. Many SD related genes such as *Vp-1/ABI3*, *MFT* /*TaPHS1*, and *Sdr*, exhibit conservative functions in *Arabidopsis*, wheat, rice, and barley [7, 8, 11]. To explore the role of *TaPer12-3A* on SD and germination in *Arabidopsis*, three independent transgenic lines (*At-L2*, *At-L6*, and *At-L9*) were generated for seed germination test (Figs. 3A and S2A). The results showed that the three overexpression lines had higher GP values (95%, 97%, and 99%, respectively) than Col-0 (GP 82%) (Table S3), indicating that *TaPer12-3A* had a negative effect on dormancy and a positive effect on enhancing germination (Fig. 3B). To further investigate the role of *TaPer12-3A* in rice SD and germination, three overexpression Nip rice lines (*35S:TaPer12-2*, *35S:TaPer12-3*, and *35S:TaPer12-5*) were tested for GP (Figs. 3C and S2B). The phenotypic data revealed that the GP values of the three independent overexpression lines were higher (95%, 91%, and 93%, respectively) than that of Nip (73%) after 7 days of imbibition (Fig. 3C-E; Table S3). These results indicated that the introduction of *TaPer12-3A* significantly improved the germination of rice seeds, implying a negative role in SD and a positive role in germination.

### Germination test of *TaPer12-3A* wheat EMS mutant seeds

**Fig. 4:**
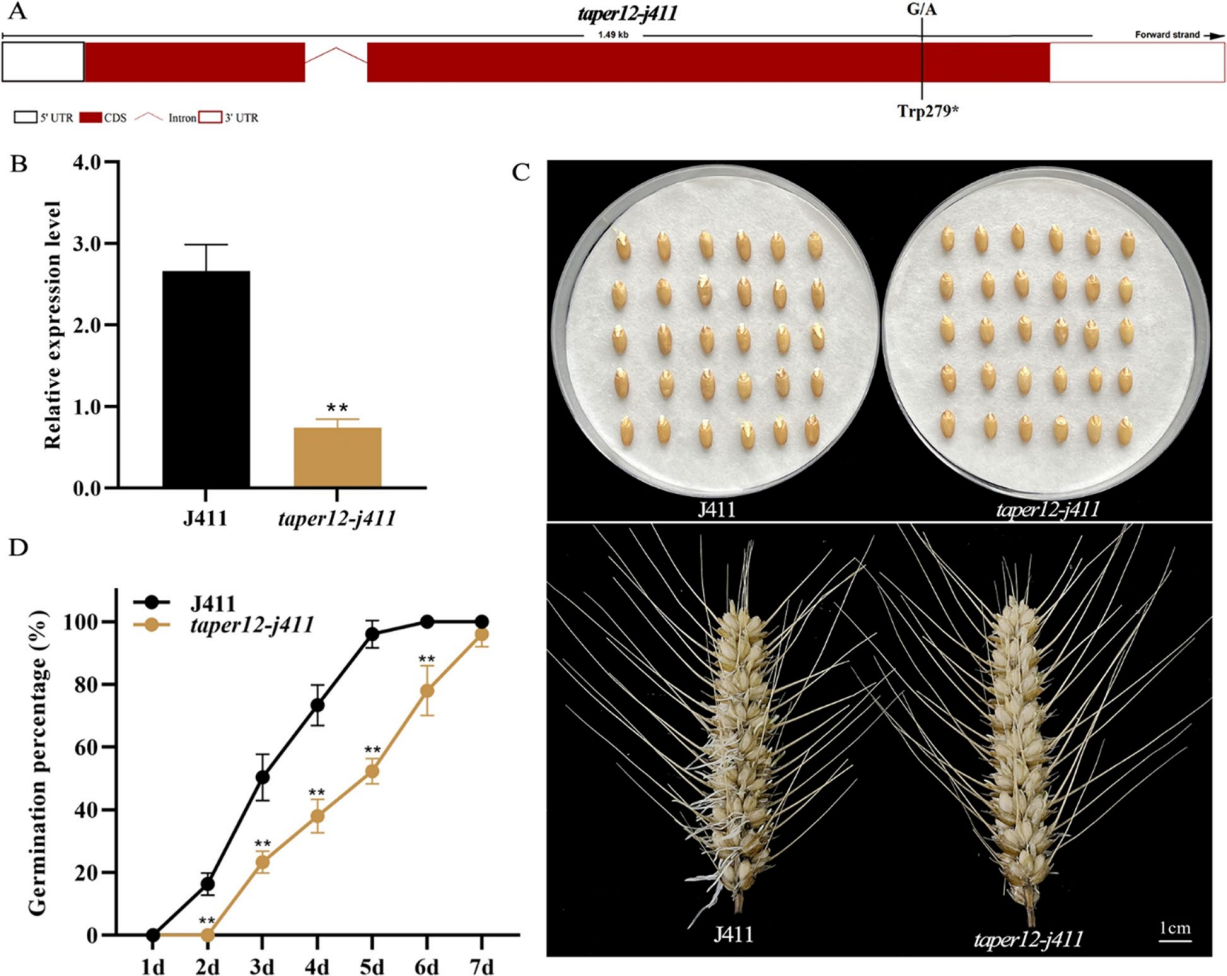
Germination tests of the seeds and spikes of the *TaPer12-3A* wheat EMS mutant. **A** Mutation site of the wheat EMS mutant *taper12-j411*. **B** Expression analysis of *TaPer12-3A* in *taper12-j411* and J411 seeds. **P < 0.01 indicates extreme significance. **C** Germination phenotypes of freshly collected *taper12-j411* and J411 seeds and spikes. The seeds and spikes germinated for 5 and 7 days, respectively. **D** Germination percentages of *taper12-j411* and J411 seeds for 7 consecutive days. **P < 0.01 indicates extreme significance

To further validate the role of *TaPer12-3A* in wheat SD and germination, we obtained the *TaPer12-3A* EMS mutant (named *taper12-j411*) in J411 background. The mutant *taper12-j411* had a single nucleotide polymorphism mutation (G/A) at the 279th amino acid in the *TaPer12-3A* functional domain (secretory POD domain), leading to early termination of translation (Fig. 4A). The relative expression level of *TaPer12-3A* was significantly lower in *taper12-j411* than in J411 (Fig. 4B). The germination test results showed that the GP value of *taper12-j411* was lower than that of wild-type J411 during seed germination (Fig. 4C and D). These results demonstrated that the mutation of *TaPer12-3A* promoted dormancy and inhibited germination, which is accordance with the results of transgenic *Arabidopsis* and rice lines.

### Possible regulatory mechanism of *TaPer12-3A* in SD and germination

**Fig. 5:**
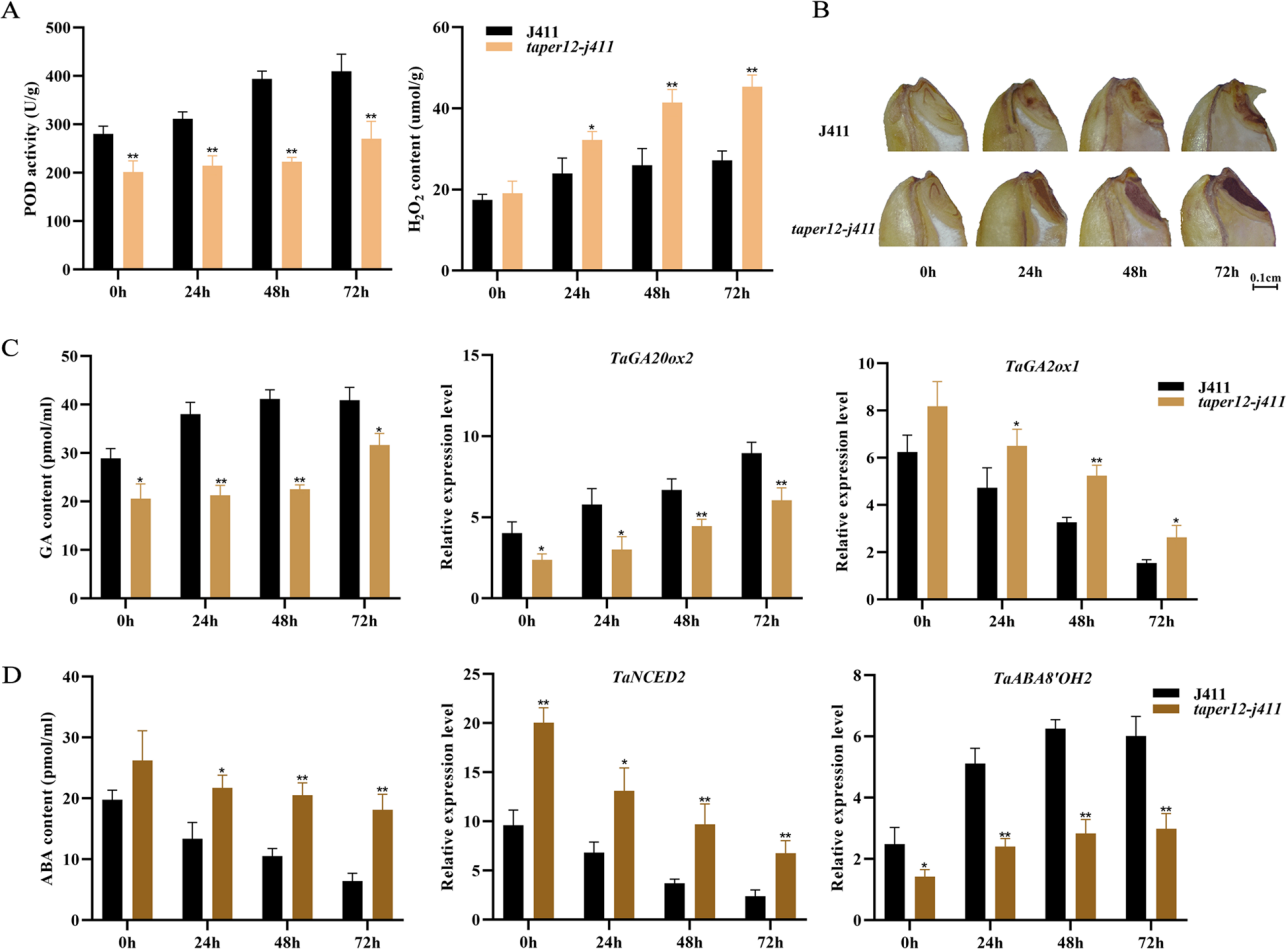
Regulatory mechanism of *TaPer12-3A* in seed dormancy and germination. **A** Hydrogen peroxide (H_2_O_2_) content and peroxidase (POD) activity measured in the wheat EMS mutant *taper12-j411* and J411 seeds. **B** Dynamic accumulation of H_2_O_2_ in *taper12-j411* and J411 seeds. **C** Gibberellic acid (GA) content, expression levels of the key genes involved in GA biosynthesis and catabolism pathways, including gene ID: *TraesCS3B02G439900* (*TaGA20ox2*) and *TraesCS1B02G123500* (*TaGA2ox1*). *P < 0.05 and **P < 0.01 indicate significance and extreme significance, respectively. **D** Abscisic acid (ABA) content, expression levels of the key genes involved in ABA biosynthesis and catabolism pathways, including gene ID: *TraesCS5A02G374000* (*TaNCED2*) and *TraesCS5D02G244900* (*TaABA8’OH2*). *P < 0.05 and **P < 0.01 indicate significance and extreme significance, respectively

To investigate how *TaPer12-3A* regulates wheat SD and germination, we measured the H_2_O_2_, GA, and ABA contents and POD activity in *taper12-j411* and J411 seeds at 0, 24, 48, and 72 h after imbibition (Table S4). POD activity was consistently lower in *taper12-j411* seeds, but H_2_O_2_ content exhibited an opposite trend (Fig. 5A). The 3,3-diaminobenzidine tetrahydrochloride (DAB) staining results showed that the embryonic parts of the *taper12-j411* seeds had higher H_2_O_2_ accumulation than that of J411 seeds during germination (Fig. 5B). The *taper12-j411* seeds had lower GA level and higher ABA level than the J411 seeds (Fig. 5C and D). During seed germination, the GA level and POD activity showed an increasing trend, while the ABA level showed a decreasing trend. Based on these data, we suggest that *TaPer12-3A* regulates wheat SD and germination through the crosstalk of H_2_O_2_, GA and ABA.

Because of significant differences in the ABA and GA levels between the *taper12-j411* and J411 seeds during germination, we further detected the expression patterns of several key genes involved in GA and ABA biosynthesis and catabolism pathways (Table S5). The key genes involved in GA biosynthesis (*TaGA20ox2*) and ABA catabolism (*TaABA8’OH2*) were downregulated in *taper12-j411* seeds compared with J411 seeds, whereas the GA catabolism gene *TaGA2ox1* and the ABA biosynthesis gene *TaNCED2* were upregulated (Fig. 5C and D). These findings support that *TaPer12-3A*, a H_2_O_2_ scavenging enzyme gene, positively regulates wheat SD release and germination by interacting with the ABA and GA pathways.

### Subcellular localization of *TaPer12-3A*

**Fig. 6:**
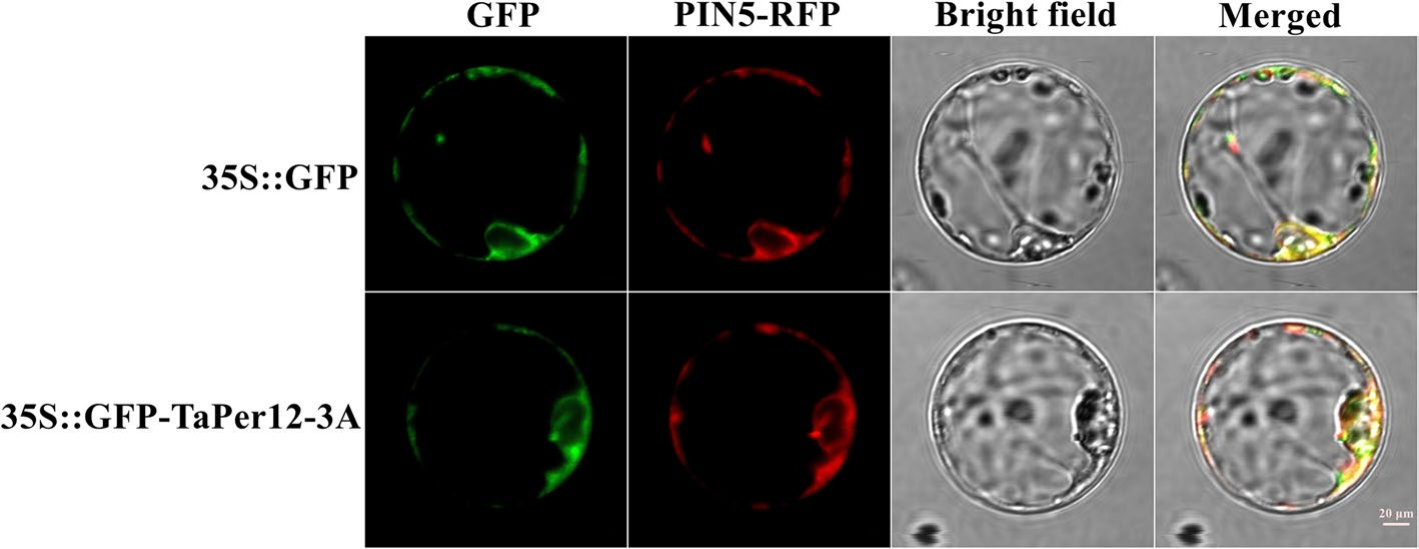
Subcellular localization of TaPer12-3A in wheat protoplast. Scale bar is 20 μm, and 35S::GFP was used as the empty control. The PIN5-RFP fusion protein served as an endoplasmic reticulum marker

To observe the subcellular distribution of TaPer12-3A, we transfected wheat protoplasts with the vector 35S::GFP-TaPer12-3A using a PEG4000-mediated technique. TaPer12-3A was present in both endoplasmic reticulum and cytoplasm (Fig. 6). The subcellular localization result helps to further dissect the molecular mechanism of *TaPer12-3A* regulating SD and germination.

## Discussion

Pre-harvest sprouting (PHS) often occurs in cereal crops such as wheat and rice. The global annual loss due to PHS is as high as $1 billion [29]. In particular, in China, in May 2023, wheat major producing regions, such as Henan and Anhui provinces, suffered severe PHS disasters. Seed dormancy and germination is closely associated with PHS. Wheat varieties with strong dormancy appears high PHS resistance. Therefore, exploring dormancy-related genes helps to breed wheat varieties with high PHS resistance.

Studies showed that an increase in POD activity often accompanies seed germination, whereas POD activity remains low during dormancy. For instance, in flower buds of potted lemon tree, POD activity did not significantly change during the winter rest period, but increased 4–fivefold upon reaching dormancy release [30]. In beetroot, POD activity was the highest during the growth stage and the lowest during the dormant period [31, 32]. In addition, during germination, the POD activity of Berberis seeds increased [33], whereas that of mung bean (*Vigna radiata*) increased initially but then decreased [34]. In sacred lotus, the expression of *NnPER1*, which encodes peroxiredoxin, was significantly elevated in the early imbibition stage. Particularly, the *NnPER1* overexpression *Arabidopsis* lines showed a high seed germination rate compared with WT [25]. The POD gene *PER1A* regulated rice seed germinability by the bZIP23*–PER1A* complex via the ABA signaling pathway, further emphasizing the role of POD in SD release and germination and its association with the ABA signaling pathway [26]. More recently, two ethylene signaling regulators, OsEIL1 and OsEIL2, directly mediated the expressions of four POD genes (*OsPRX37*, *OsPRX81*, *OsPRX82*, and *OsPRX88*) and facilitated rice coleoptile growth by scavenging ROS [35]. However, the specific function of POD in regulating wheat SD and germination remains to be elucidated.

In the present study, to investigate the function of *TaPer12-3A* encoding a class III POD in regulating wheat SD and germination, we first detected the expression patterns of *TaPer12-3A* during dormancy acquisition and release in two wheat varieties with contrasting SD and germination phenotypes (HMC21 and J411). The expression level of *TaPer12-3A* was consistently higher in J411 seeds (weak SD) than in HMC21 seeds (strong SD), implying that *TaPer12-3A* may promote SD release and germination. We heterologously overexpressed *TaPer12-3A* in rice and *Arabidopsis* and found that the germination rate was higher in transgenic seeds than in WT seeds. Finally, using the EMS mutant of J411, we found that the loss-of-function of *TaPer12-3A* decreased the seed germination rate and increased dormancy level, indicating a negative role of *TaPer12-3A* in dormancy and a positive role in elevating germinability. In particular, the mutant *taper12-j411* showed no obvious defects in other yield-related traits, which may be an excellent germplasm resource for the genetic improvement of wheat PHS resistance (Fig. S9). Next, we will perform transgenic experiments in a wheat background to confirm the function of *TaPer12-3A*.

In plants, class III POD is a crucial enzyme for efficient scavenging of ROS (H_2_O_2_). Liu et al. [18] reported that the regulation of *Arabidopsis* SD and germination by H_2_O_2_ was achieved by influencing the ABA catabolism and GA biosynthesis pathways. Wang et al. [26] found that the rice POD gene *PER1A* regulated seed germinability by the bZIP23*–PER1A* complex, which involves the ABA signaling pathway. To explore the regulatory mechanism of *TaPer12-3A* in SD and germination, we not only measured the H_2_O_2_, GA, ABA and contents and POD activity, but also detected the expression patterns of several key genes involved in GA and ABA biosynthesis and catabolism pathways in *taper12-j411* and J411 seeds. These results confirmed that *TaPer12-3A* increased POD activity and participated in the crosstalk of GA and ABA signaling pathways, thereby mediating SD and germination, which is similar to the results reported by Wang et al. [26]. To play a positive role in seed germination, TaPer12-3A can maintain H_2_O_2_ in a certain concentration range, which is called the “oxidative window” [36]. Only at this suitable H_2_O_2_ concentration can the expression of GA biosynthesis genes and ABA catabolism genes be enhanced to promote seed germination [18, 37, 38]. In addition, class III peroxidases are a family of polygenic secreted enzymes that are localized in all plant organs [39]. At the protein level, the membrane localization of class III peroxidases has been shown to be applicable to different plants [40]. In this study, we also investigated the subcellular localization of TaPer12-3A and found that TaPer12-3A was present in endoplasmic reticulum and cytoplasm. These findings will lay the foundation for an in-depth analysis of molecular mechanism of *TaPer12-3A* in regulating wheat seed dormancy. In summary, our findings are useful for further understanding the role of *TaPer12-3A* in wheat SD and germination.

## Conclusion

By combining transcriptome data and expression analysis, we identified a class III POD gene, *TaPer12-3A*, associated with SD and germination. Using overexpression *Arabidopsis* and rice lines and wheat EMS mutant plants, we confirmed a positive role of *TaPer12-3A* in SD release and germination. Moreover, *TaPer12-3A* maintains H_2_O_2_ homeostasis by scavenging excess H_2_O_2_ and participates in the GA and ABA pathways to promote seed germination. These results not only provide a novel gene resource for breeding wheat varieties with high SD and PHS resistance by the gene editing approach but also elucidate the role of POD in regulating wheat SD and germination.

## Materials and methods

### Plant materials and growth conditions

Seed samples of wheat landrace Waitoubai (WTB) with strong SD were collected at different growth stages (14, 21, 28, and 35 DPA) and at different post-ripening stages (7, 14, 21, and 28 DAH) for expression analysis of *TaPer12-3A*. To investigate the expression patterns of *TaPer12-3A*, the seeds of two wheat varieties with contrasting SD phenotypes, Jing 411 (J411) with weak SD and Hongmangchun 21 (HMC21) with strong SD, were sampled at 1, 6, 9, 12, and 36 h after seed imbibition. WTB, J411, and HMC21 were grown in the experimental plot during the 2021–2022 cropping season (Hefei, China). Three biological replicates were performed for each test sample, and these were stored at − 80 °C. The seeds of J411, HMC21 and WTB were provided by Key Laboratory of Wheat Biology and Genetic Improvement on Southern Yellow & Huai River Valley.

The mutant (*taper12-j411*, 3A_731157375_E) mutagenized with EMS in a J411 background was used to validate the role of *TaPer12-3A* in SD and germination. The EMS mutant of the M_5_ generation used to assess germination phenotypes was grown in a greenhouse at 23 ± 1 °C (16 h light/8 h dark, 60% relative humidity). The EMS mutant was provided by Boruidi Biotechnology Co., Ltd., Shijiazhuang, Hebei, China (http://www.molbreeding.com).

The *TaPer12-3A* coding sequence was amplified from WTB cDNA and inserted into the pCAMBIA1301a (p1301) binary vector using *EcoR* I and *Bam* HI sites under the control of the constitutive cauliflower mosaic virus 35S (CaMV35S) promoter (GenBank accession number PP532809, https://www.ncbi.nlm.nih.gov/nuccore/PP532809.1) (Figs. S3 and S4). The cDNA of *TaPer12-3A* was amplified from wheat variety (WTB) by PCR and inserted into the pCAMBIA1301a vector with double CaMV35S promoters. The recombinant plasmid was transfected into Agrobacterium *tumefaciens* GV3101 and introduced into wild type (WT) *Arabidopsis thaliana* (Columbia, Col-0) using the floral dip method. The seeds of T_0_ transgenic plants were selected on 1/2 MS medium (pH 5.8) containing 50 mg/L hygromycin. For rice transformation, the constructions were introduced into A. *tumefaciens* strain EHA105, and then transformed into rice calli through an Agrobacterium-mediated strategy. To verify whether the plants were positive plants, we extracted DNA from T_1_ overexpression and WT (*Oryza sativa* L.) *Japonica* (Nipponbare, Nip) plants (WT as the negative control) (Fig. S8; Table S1). In addition, we detected the expression level of *TaPer12-3A* in T_1_ overexpression lines by qRT-PCR, and selected the lines with the highest relative expression level of *TaPer12-3A* to grow into T_2_ generation (Fig. S2). The seeds of Col-0 and Nip were provided by Key Laboratory of Wheat Biology and Genetic Improvement on Southern Yellow & Huai River Valley.

The WT *Arabidopsis* Col-0 and overexpression lines were vernalized for 3 days at 4 °C, moved to a 24 ± 1 °C greenhouse (16 h light/8 h dark) for 7 days, and then moved to square planting pots containing a mixture of vermiculite and black soil (3:1, v/v).

The WT rice Nip and overexpression lines were grown in a greenhouse (28 °C day/25 °C night; 10 h light/14 h dark), with humidity levels maintained at approximately 70% and light intensity at 200 μmol photons m^−2^ s^−1^.

### Germination phenotype assay

GP was used to evaluate SD in wheat seeds. The intact and healthy wheat seeds were divided into three replicates and placed in a culture dish with a diameter of 90 mm. The filter paper was moistened with 9 mL distilled water, and incubated at 20 °C with a photoperiod of 14 h day/10 h night and a relative humidity of 80%. Germination was defined as visible rupture of the pericarp and testa [41]. In the germination test, GP was recorded regularly on a daily basis. GP was the number of germinated seeds divided by the total number of viable seeds.

For the germination test of *Arabidopsis* seeds, seeds were placed on two layers of filter paper, which were wetted with deionized water, and grown in a growth chamber (23 °C day/21 °C night, 16 h light/8 h dark). GP of *Arabidopsis* seeds was assessed on the 7th day. The presence of a protruding radicle was deemed positive germination [42].

For the rice seed germination test, seeds were placed on two layers of filter paper moistened with deionized water, and subsequently cultivated in a growth chamber (28 °C day/25 °C night, 10 h light/14 h dark). GP of rice seeds was assessed on the 7th day. Seeds were considered to have successfully germinated if the radicle length was ≥ 1 mm [43].

For germination assays of rice panicles and wheat spikes, at least five freshly harvested rice panicles and wheat spikes (35 DPA) were immersed in deionized water and were vertically placed in constant temperature incubators at 25 °C and 20 °C, respectively. Wheat spikes and rice panicles were transferred to fresh water every day [44]. Photographs of wheat spikes and rice panicles were taken 7 days after treatment.

### Cloning and sequence analysis of *TaPer12-3A*

The full-length genomic sequence of *TaPer12-3A* was obtained from IWGSC RefSeq v.1.0. Clone primers were designed using Primer Premier v.5.0 for cloning the full-length genomic sequence of *TaPer12-3A* in WTB. Polymerase chain reaction (PCR) was performed using Phanta Max Super-Fidelity DNA polymerase (Vazyme Biotech, Nanjing, China) in a 20-µL mixed reaction system. The reaction was carried out in a T100 Thermal Cycler (Bio-Rad, USA) with the following procedure: denaturation at 95 °C for 3 min, followed by 36 cycles at 95 °C for 15 s, 60 °C for 15 s, and 72 °C for 20 s, and a final extension at 72 °C for 5 min. The PCR products were assayed on a 1.2% agarose gel, and the target fragments were excised from the gel and purified using the FastPure Gel DNA Extraction Mini Kit (Vazyme Biotech). The PCR products were ligated into TA-cloning vector (CB501-2, TransGen Biotech) and transformed into E. coli (CD501, TransGen Biotech). Three target fragments were selected for sequencing analysis (Sangon Biotech, Shanghai, China) (Table S1).

Sequence homology was determined by BLAST comparison with the NCBI database (https://www.ncbi.nlm.nih.gov/). Protein sequences from different plants with the highest homology to TaPer12 were retrieved from the GenBank database, and the phylogenetic tree was constructed using MEGA v.7.0 (https://www.megasoftware.net/). Multiple sequences of Per12 were analyzed using DNAMAN software.

### Expression analysis of *TaPer12-3A*

We analyzed RNA sequencing data of WTB treated with high temperature during development in our previous study [27]. From 21 to 35 DPA, wheat landrace WTB with strong SD was treated with high temperature. The seed samples were collected at three developmental stages (21, 28, and 35 DPA) under normal (25 °C day/20 °C night) and high temperature (35 °C day/25 °C night) conditions and used for transcriptome sequencing (https://www.ncbi.nlm.nih.gov/bioproject/PRJNA895954). The expression patterns of *TaPer12-3A* in different tissues were investigated by analyzing the transcriptome data of wheat variety ‘Chinese Spring’ (http://202.194.139.32).

### RNA extraction and qRT-PCR analyses

The RNA Extraction Kit (AG21017; Accurate Bio Inc., Hunan, China) was used to extract total RNA from seeds. qRT-PCR primers were designed using Primer Premier (Table S1). We used the *TaActin* and *OsActin* genes as internal control genes for normalization [45]. Reverse transcription was performed using the HiScript II Q RT SuperMix for qPCR (+ gDNA wiper) (R223-01; Vazyme Biotech) in a 10-μL reaction system. We used a two-step program with the Applied Biosystems 7500 Real-Time PCR System (Bio-Rad Laboratories, USA) for qRT-PCR, consisting of a 90 °C denaturation step for 30 min, followed by 30–40 cycles at 95 °C for 30 s, 60 °C for 15 s, and 72 °C for 10 s. After data processing, we generated the corresponding graph using GraphPad Prism v.8 (GraphPad Software, Boston, MA, USA) and used the 2^−ΔΔCt^ method to determine the relative gene expression [46].

### Measurement of GA, ABA, and H_2_O_2_ contents and POD activity

Wheat seeds were sampled at 24, 48, and 72 h after imbibition in a growth chamber (16 h light/8 h dark, 25 ± 1 °C), and used to determine the GA, ABA, and H_2_O_2_ contents and POD activity. The wheat seeds were sampled at 48 h after imbibition in a growth chamber (16 h light/8 h dark, 23 ± 1 °C). ELISA kits (MM-013801 and MM-012501; Jiangsu Meimian Industrial Co., Ltd., China) were used to measure ABA and GA contents. Kits (BC3590 and BC0090; Solarbio, Beijing, China) were used to test H_2_O_2_ content and POD activity following the manufacturer’s instructions.

### Histochemical detection of H_2_O_2_

H_2_O_2_ can react rapidly with 3,3-diaminobenzidine tetrahydrochloride (DAB) to form a brownish-red compound, thus locating H_2_O_2_ in tissues. Seeds of J411 and *taper12-j411* imbibed for different periods were divided longitudinally into two parts, immersed in DAB solution at 25 °C for 6 h, and then photographed.

### Subcellular localization of TaPer12-3A

To investigate the subcellular localization of the TaPer12-3A protein, we used the p1301 vector containing a reporter gene encoding green fluorescent protein (GFP) [47]. A control was created by constructing a p1301-CaMV35S-TaPer12-GFP expression vector using primers that included *Xba* I and *Bam* HI restriction sites, with the stop codon being excluded from the GFP reporter gene sequence (Wuhan BioRun Biosciences Co., Ltd.) (Figs. S6 and S7B). Wheat protoplasts were obtained from J411 seedlings. About 1 g of leaves was sliced into 0.5-mm strips and incubated in a 10-mL enzyme solution. Afterward, the protoplasts were rinsed twice with W5 (NaCl, CaCl_2_, KH_2_PO_4_, MES, glucose) solution. A plasmid (10 μg) and 40% PEG4000 were mixed with the protoplasts using a blender (co-localization with additional 10-μL marker co-transfection). The protoplasts were cultured in W5 solution for 10–15 h in the dark. Finally, the fluorescence signal was detected using laser confocal microscopy [48].

### Statistical analysis

The statistical analysis of the data was performed using GraphPad Prism v.8 software. We calculated the mean and standard deviation values from nine measurements, which consisted of three biological replicates, each containing three technical replicates. Student’s *t*-tests were used to compare the mean values of treatment and control plants to identify any statistically significant differences. We used a significance threshold of *P < 0.05 and **P < 0.01 to determine the statistical significance of the results.

### Supplementary Information

**Supplementary Material 1.**

## Data Availability

All data related to this manuscript can be found within this paper and its supplementary data. TaPer12-3A gene sequence during the current study are available in the [National Center for Biotechnology Information] repository, [GenBank accession number is PP532809].

## Abbreviations

POD: Peroxidases
SD: Seed dormancy
EMS: Ethyl methane sulfonate
PHS: Pre-harvest sprouting
ROS: Reactive oxygen species
H_2_O_2_: Hydrogen peroxide
GA: Gibberellic acid
ABA: Abscisic acid
GP: Germination percentage
DPA: Days post-anthesis
DAH: Days after harvest
DAB: 3,3-Diaminobenzidine tetrahydrochloride
GFP: Green fluorescent protein

## References

[1] Gelin JR, Elias EM, Kianian SF (2006). Evaluation of two durum wheat (*Triticum turgidum* L. var. *durum*) crosses for pre-harvest sprouting resistance. Field Crops Res.

[2] Liu SB, Cai SB, Graybosch R, Chen CX, Bai GH (2008). Quantitative trait loci for resistance to pre-harvest sprouting in US hard white winter wheat Rio Blanco. Theor Appl Genet.

[3] Amano Y, Torada A (2002). Breeding of white-grained wheats for japan. Euphytica.

[4] Mares DJ, Rathjen J, Mrva K, Cheong J (2009). Genetic and environmental control of dormancy in white-grained wheat. Euphytica.

[5] Ji T, Bryan P, Byung-Kee B (2018). Pre-harvest sprouting resistance of soft winter wheat varieties and associated grain characteristics. J Cereal Sci.

[6] Rajjou L, Duval M, Gallardo K, Catusse J, Bally J, Job C, Job D (2012). Seed germination and vigor. Annu Rev Plant Biol.

[7] Yang Y, Zhao XL, Xia LQ, Chen XM, Xia XC, Yu Z, He H, Roder M (2007). Development and validation of a *viviparous-1* STS marker for pre-harvest sprouting tolerance in Chinese wheat. Theor Appl Genet.

[8] Nakamura S, Abe F, Kawahigashi H, Akazono K, Tagiri A, Matsumoto T, Utsugi S, Ogawa T, Handa H, Ishida H, Mori M, Kawaura K, Ogihara Y, Miura H (2011). A wheat homolog of *MOTHER OF FT AND TFL1* acts in the regulation of germination. Plant Cell.

[9] Ashikawa I, Mori M, Nakamura S, Abe F (2014). A transgenic approach to controlling wheat seed dormancy level by using Triticeae *DOG1-*like genes. Transgenic Res.

[10] Torada A, Koike M, Ogawa T, Takenouchi Y, Tadamura K, Wu JZ, Matsumoto T, Kawaura K, Ogihara Y (2016). A causal gene for seed dormancy on wheat chromosome 4A encodes a MAP kinase kinase. Curr Biol.

[11] Zhang YJ, Xia XC, He ZH (2017). The seed dormancy allele *TaSdr-A1a* associated with pre-harvest sprouting tolerance is mainly present in Chinese wheat landraces. Theor Appl Genet.

[12] Wei WX, Min XY, Shan SY, Jiang H, Cao JJ, Li L, Wang JF, Wang SX, Zhu YL, Lu J, Si HQ, Xia XC, Ma CX, Zhang HP, Chang C (2019). Isolation and characterization of *TaQsd1* genes for period of dormancy in common wheat (*Triticum aestivum* L.). Mol Breed.

[13] Lang J, Fu YX, Zhou Y, Cheng MP, Deng M, Li ML, Zhu TT, Yang J, Guo XJ, Gui LX (2021). *Myb10-D* confers *PHS-3D* resistance to pre-harvest sprouting by regulating NCED in ABA biosynthesis pathway of wheat. New Phytol.

[14] Tai L, Wu JH, Jing YX, Liu HZ, Zeng QD, Xu XJ, Shi S, Wang HJ, Liu WT, Sun JQ, Han DJ, Chen KM (2023). A genome-wide association study uncovers that *TaPI4K-2A* regulates pre-harvest sprouting in wheat. Plant Commun.

[15] El-Maarouf-Bouteau H, Bailly C (2008). Oxidative signaling in seed germination and dormancy. Plant Signal Bechav.

[16] Weitbrecht K, Müller K, Leubner-Metzger G (2011). First off the mark: early seed germination. J Exp Bot.

[17] Law SR, Narsai R, Whelan J (2014). Mitochondrial biogenesis in plants during seed germination. Mitochondrion.

[18] Liu Y, Ye N, Rui L, Chen M, Zhang J (2010). H_2_O_2_ mediates the regulation of ABA catabolism and GA biosynthesis in *Arabidopsis* seed dormancy and germination. J Exp Bot.

[19] Bahin E, Bailly C, Sotta B, Kranner I, Corbineau F, Leymarie J (2011). Crosstalk between reactive oxygen species and hormonal signalling pathways regulates grain dormancy in barley. Plant Cell Environ.

[20] Zhang Y, Chen B, Xu Z, Shi Z, Chen S, Huang X, Cheng JX, Wang XF (2014). Involvement of reactive oxygen species in endosperm cap weakening and embryo elongation growth during lettuce seed germination. J Exp Bot.

[21] Ishibashi Y, Yamamoto K, Tawaratsumida T, Yuasa T, Iwaya-Inoue M (2008). Hydrogen peroxide scavenging regulates germination ability during wheat (*Triticum aestivum* L.) seed maturation. Plant Signal Bechav.

[22] Shigeto J, Tsutsumi Y (2016). Diverse functions and reactions of class III peroxidases. New Phytol.

[23] Sorokan AV, Burhanova GF, Maksimov IV (2018). Anionic peroxidase-mediated oxidative burst requirement for jasmonic acid-dependent *Solanum tuberosum* defence against *Phytophthora infestans*. Plant Pathol.

[24] Kidwai M, Dhar YV, Gautam N, Tiwari M, Ahmad IZ, Asif MH, Chakrabarty D (2019). Oryza sativa class III peroxidase (*OsPRX38*) overexpression in *Arabidopsis thaliana* reduces arsenic accumulation due to apoplastic lignification. J Hazard Mater.

[25] Chen HH, Chu P, Zhou YL, Ding Y, Li Y, Liu J, Jiang LW, Huang SZ (2016). Ectopic expression of NnPER1, a *Nelumbo nucifera* 1-cysteine peroxiredoxin antioxidant, enhances seed longevity and stress tolerance in *Arabidopsis*. Plant J.

[26] Wang WQ, Xu DY, Sui YP, Ding XH, Song XJ (2022). A multiomic study uncovers a *BZIP23-PER1A*-mediated detoxification pathway to enhance seed vigor in rice. Proc Natl Acad Sci USA.

[27] Jiang H, Gao W, Jiang BL, Liu X, Jiang YT, Zhang LT, Zhang Y, Yan SN, Cao JJ, Lu J, Ma CX, Chang C, Zhang HP (2023). Identification and validation of coding and non-coding RNAs involved in high-temperature-mediated seed dormancy in common wheat. Front Plant Sci.

[28] Consortium TIWGS (2014). A chromosome-based draft sequence of the hexaploid bread wheat (*Triticum aestivum*) genome. Science.

[29] Vetch JM, Stougaard RN, Martin JM, Giroux MJ (2019). Review: revealing the genetic mechanisms of pre-harvest sprouting in hexaploid wheat (*Triticum aestivum* L.). Plant Sci.

[30] Kasraoui MF, Duquesnoy I, Winterton P, Lamaze T (2014). Soluble and cell wall bound peroxidase activities are markers of flower bud development stages in lemon (*Citrus limon* L.). J Appl Bot Food Qual.

[31] Nimaeva OD, Pradedova EV, Salyaev RK (2014). Activity and isoenzyme composition of vacuolar peroxidasein the roots of red beet at different stages of development and upon changes in storage conditions. Russ J Plant Physiol.

[32] Pradedova EV, Nimaeva OD, Salyaev RK (2014). Effect of stress conditions on the activity and isozyme composition of peroxidase of vacuoles and tissue extract of red beet roots. Biol Bull.

[33] Belwal T, Bisht A, Bhatt ID, Rawal RS (2015). Influence of seed priming and storage time on germination and enzymatic activity of selected berberis species. Plant Growth Regul.

[34] Singh HP, Kaur S, Batish DR, Kohli RK (2014). Ferulic acid impairs rhizogenesis and root growth, and alters associated biochemical changes in mung bean (*Vigna radiata*) hypocotyls. J Plant Interact.

[35] Qiao JZ, Quan RD, Wang J, Li YX, Xiao DL, Zhao ZH, Huang RF, Qin H (2024). OsEIL1 and OsEIL2, two master regulators of rice ethylene signaling, promote the expression of ROS scavenging genes to facilitate coleoptile elongation and seedling emergence from soil. Plant Commun.

[36] Bailly C, El-Maarouf-Bouteau H, Corbineau F (2008). From intracellular signaling networks to cell death: the dual role of reactive oxygen species in seed physiology. C R Biol.

[37] Parkhey S, Naithani SC, Keshavkant S (2012). ROS production and lipid catabolism in desiccating Shorea robusta seeds during aging. Plant Physiol Biochem.

[38] Gomes MP, Garcia QS (2013). Reactive oxygen species and seed germination. Biologia.

[39] Passardi F, Theiler G, Zamocky M, Cosio C, Rouhier N, Teixera F, Margis-Pinheiro M, Dunand C (2007). PeroxiBase: the peroxidase database. Phytochemistry.

[40] Sabine L, Martinez-Cortes T (2019). Molecular sciences membrane-bound class III peroxidases: unexpected enzymes with exciting functions. Int J Mol Sci.

[41] Zuo JH, Lin CT, Cao H, Chen FY, Liu YX, Liu JD (2019). Genome wide association study and quantitative trait loci mapping of seed dormancy in common wheat (*Triticum aestivum* L.). Planta.

[42] Cao H, Han Y, Li J, Ding M, Li Y, Li XY, Chen FY, Soppe WJ, Liu YX (2020). Arabidopsis thaliana SEED DORMANCY 4-LIKE regulates dormancy and germination by mediating the gibberellin pathway. J Exp Bot.

[43] Li QF, Zhou Y, Xiong M, Ren XY, Han L, Wang JD, Zhang CQ, Fan XL, Liu QQ (2020). Gibberellin recovers seed germination in rice with impaired brassinosteroid signalling. Plant Sci.

[44] Xu F, Tang JY, Wang SX, Cheng X, Wang HR, Ou SJ, Gao SP, Li BS, Qian YW, Gao CX, Chu CC (2022). Antagonistic control of seed dormancy in rice by two bHLH transcription factors. Nat Genet.

[45] Bai JF, Wang YK, Guo LP, Guo XM, Guo HY, Yuan SH, Duan WJ, Liu Z, Zhao CP, Zhang FT, Zhang LP (2019). Genomic identification and characterization of MYC family genes in wheat (*Triticum aestivum* L.). BMC Genom.

[46] Livak KJ, Schmittgen TD (2001). Analysis of relative gene expression datausing real-time quantitative PCR and the 2^−ΔΔCT^ method. Methods.

[47] Nelson BK, Cai X, Nebenführ A (2007). A multicolored set of in vivo organelle markers for co-localization studies in *Arabidopsis* and other plants. Plant J.

[48] Zhang Y, Su J, Duan S, Ao Y, Dai J, Liu J, Wang P, Li Y, Liu B, Feng D, Wang J, Wang H (2011). A highly efficient rice green tissue protoplast system for transient gene expression and studying light/chloroplast-related processes. Plant Methods.

