# Supplementary material for: Functional analysis of a wheat class III peroxidase gene, TaPer12-3A, in seed dormancy and germination
Source: BMC Plant Biol. 2024 Apr 24;24:318. doi: 10.1186/s12870-024-05041-4 (PMC11040755; doi:10.1186/s12870-024-05041-4)
Supplement: Supplementary file 1 — Supplementary Material 1. [file 12870_2024_5041_MOESM1_ESM.doc]

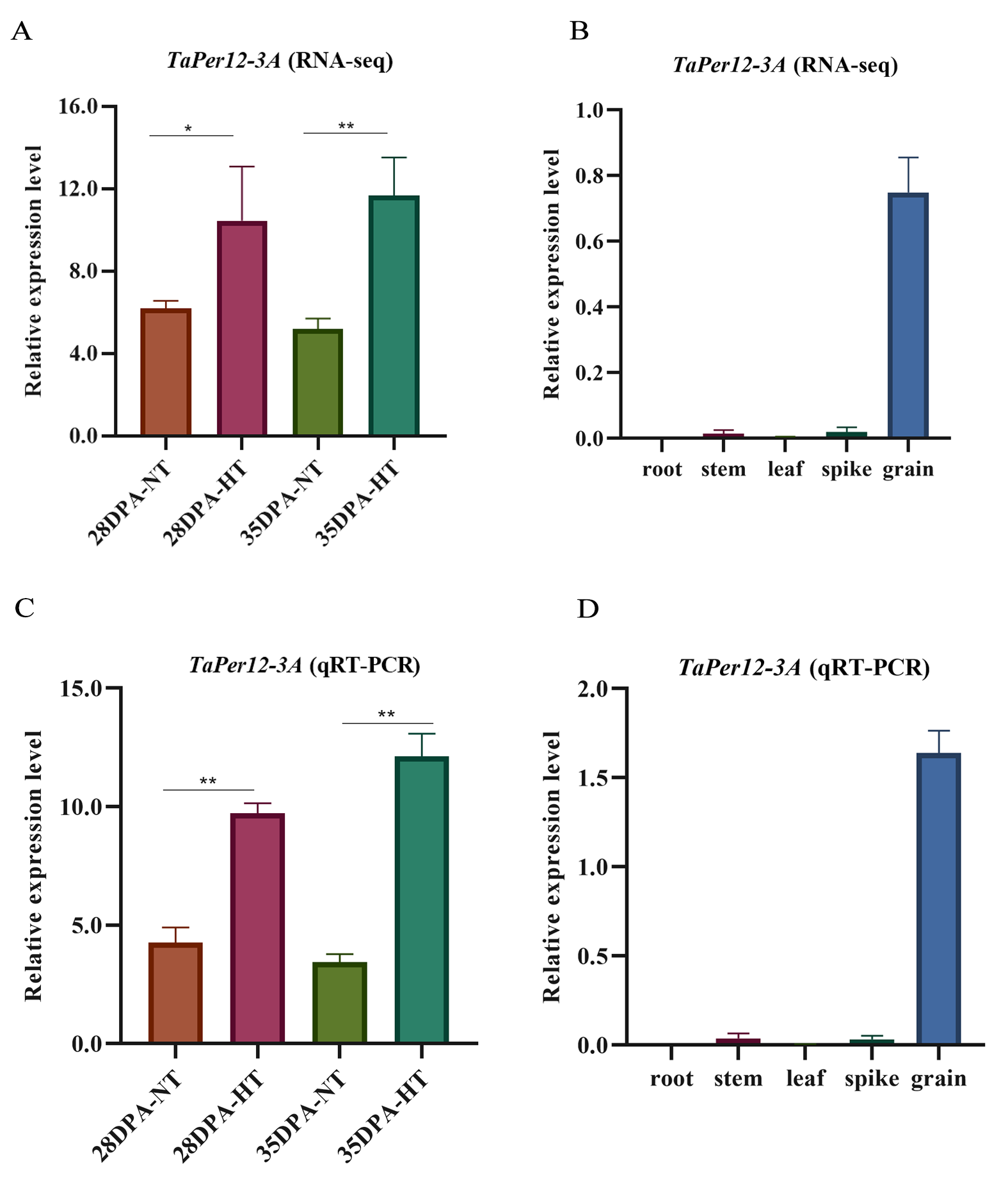


**Fig. S1. Relative expression of *TaPer12-3A* by qRT-PCR.**

**A**. Relative expression of *TaPer12-3A* in Waitoubai (WTB) seeds treated with high temperature by transcriptome sequencing. **B**. Relative expression of *TaPer12-3A* in different wheat tissues from the public expression database. **C**. Relative expression of *TaPer12-3A* in WTB seeds treated with high temperature by qRT**-**PCR. **D**. Relative expression of *TaPer12-3A* in different wheat tissues by qRT**-**PCR. ***P* < 0.01 indicates extreme significance.


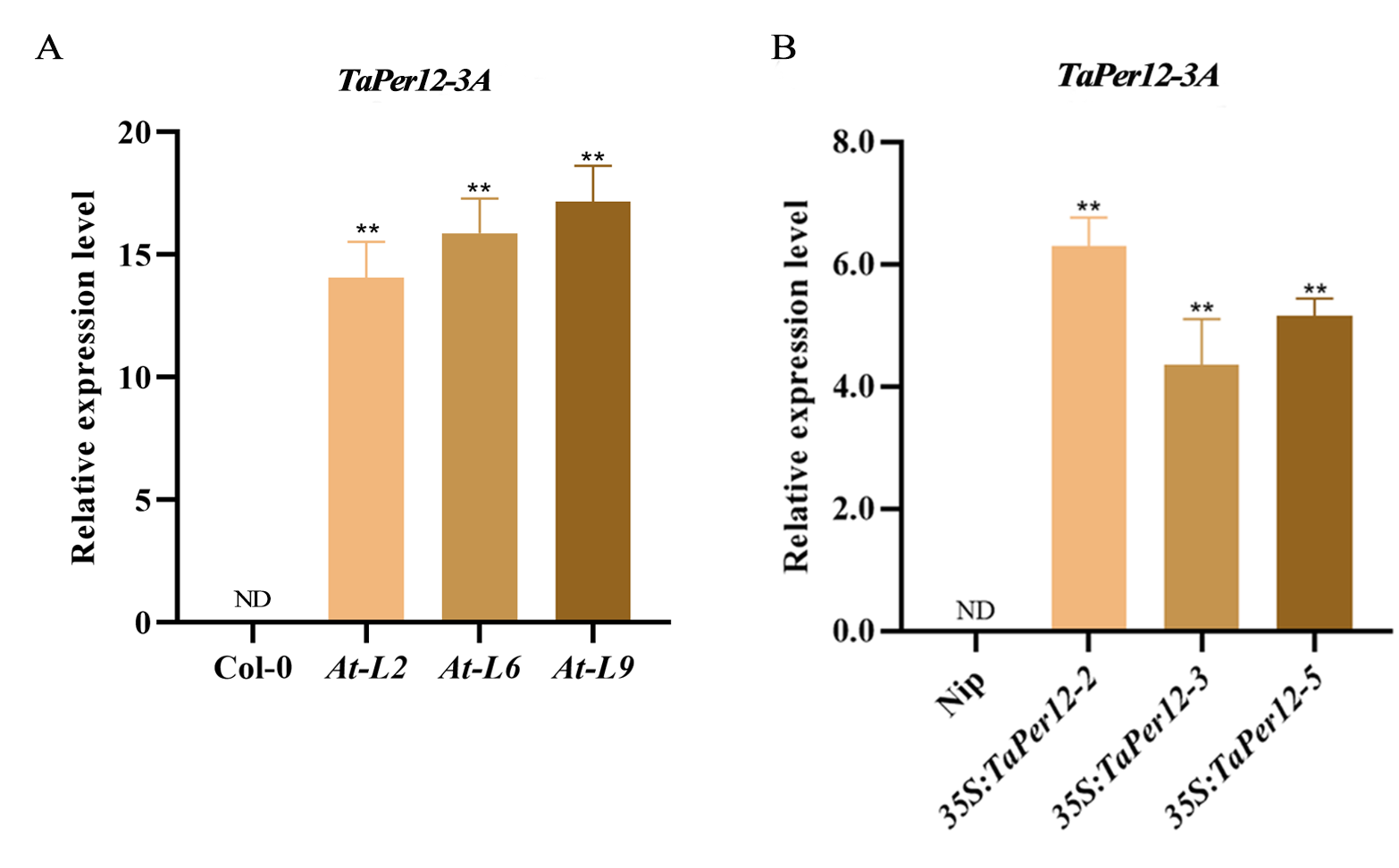


**Fig. S2. Relative expression of *TaPer12-3A* in overexpression *Arabidopsis* and rice seeds.**

**A**. Relative expression of *TaPer12-3A* in overexpression *Arabidopsis* (*At-L2/-6/-9*) and Col-0 seeds. RNA was extracted from seeds imbibed for 24 h. ND represents not detected. **B**. Expression patterns of *TaPer12-3A* in overexpression rice (*35S:TaPer12*) and Nipponbare (Nip) seeds imbibed for 24 h. ND indicates *TaPer12-3A* not detected. ***P* < 0.01 indicates extreme significance.


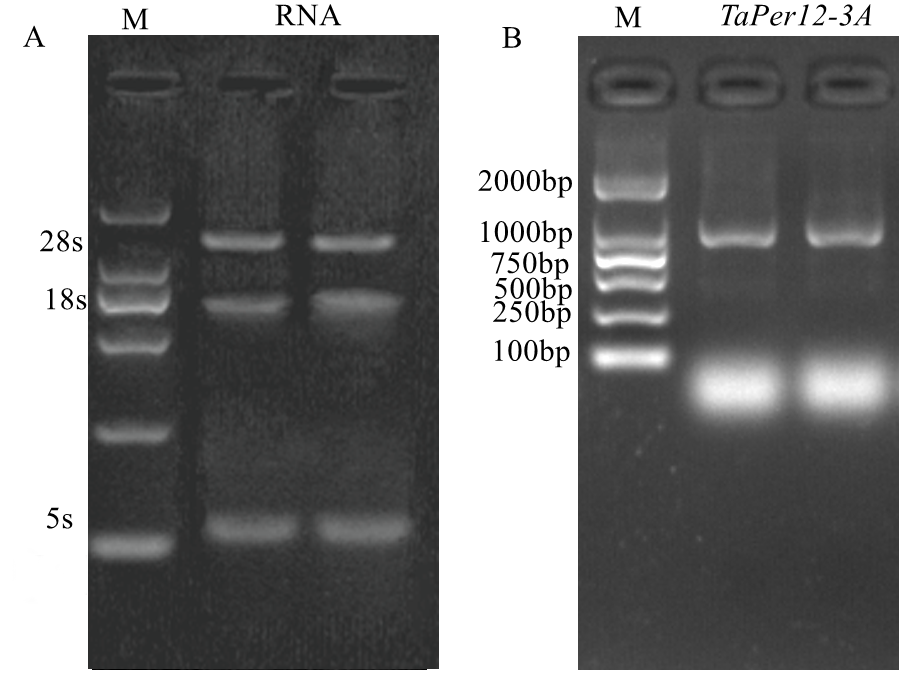


**Fig. S3. The *TaPer12-3A* vector DNA preparation**

**A**. Detection of total RNA extracted from the seeds of wheat variety Waitoubai (WTB). **B**. The gel image of *TaPer12-3A* cDNA.


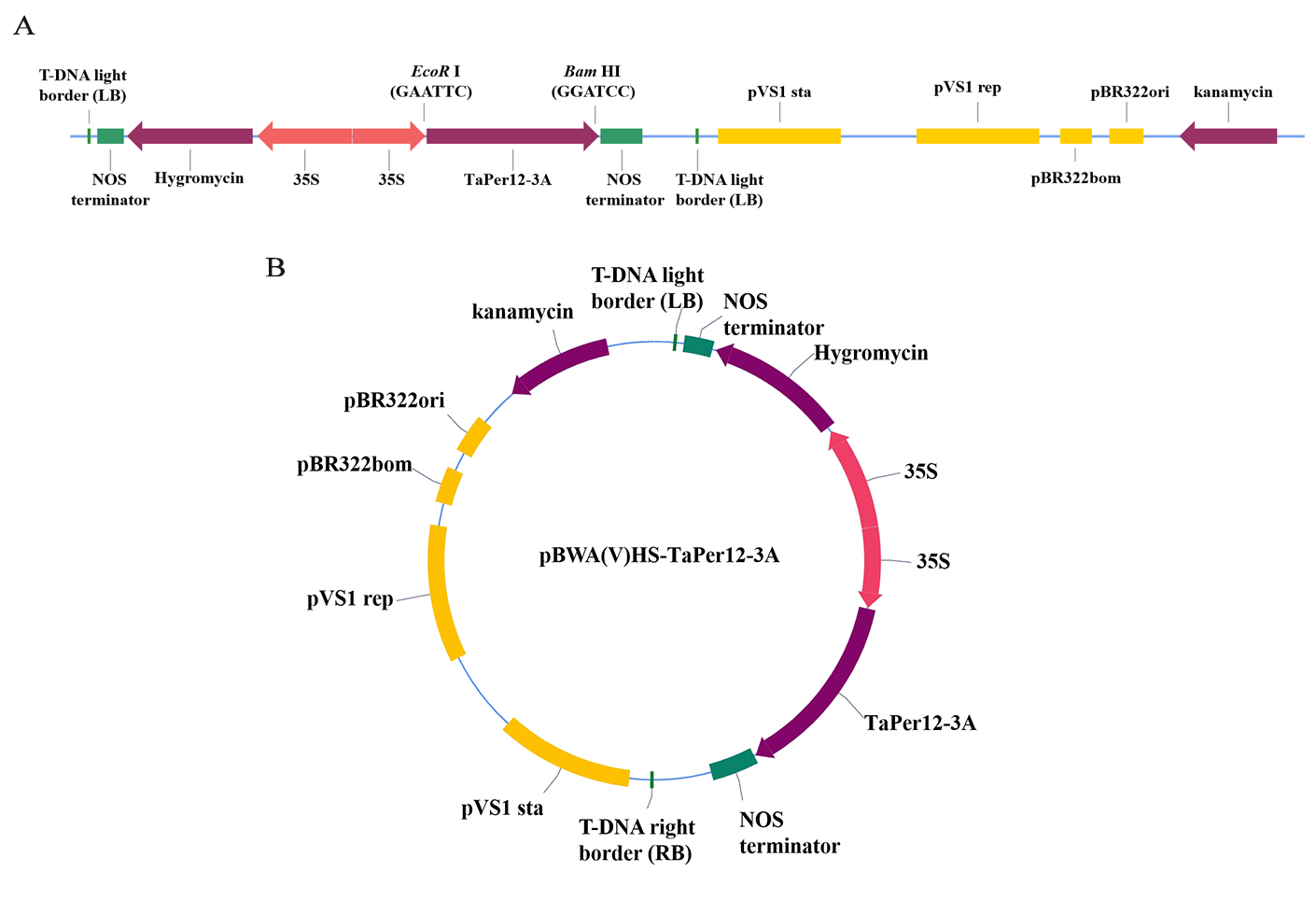


**Fig. S4. Overexpression vector mapping of the *TaPer12-3A* gene**

**A**. Line map of the *TaPer12-3A* gene overexpression vector. **B**. Loop map of the *TaPer12-3A* gene overexpression vector.


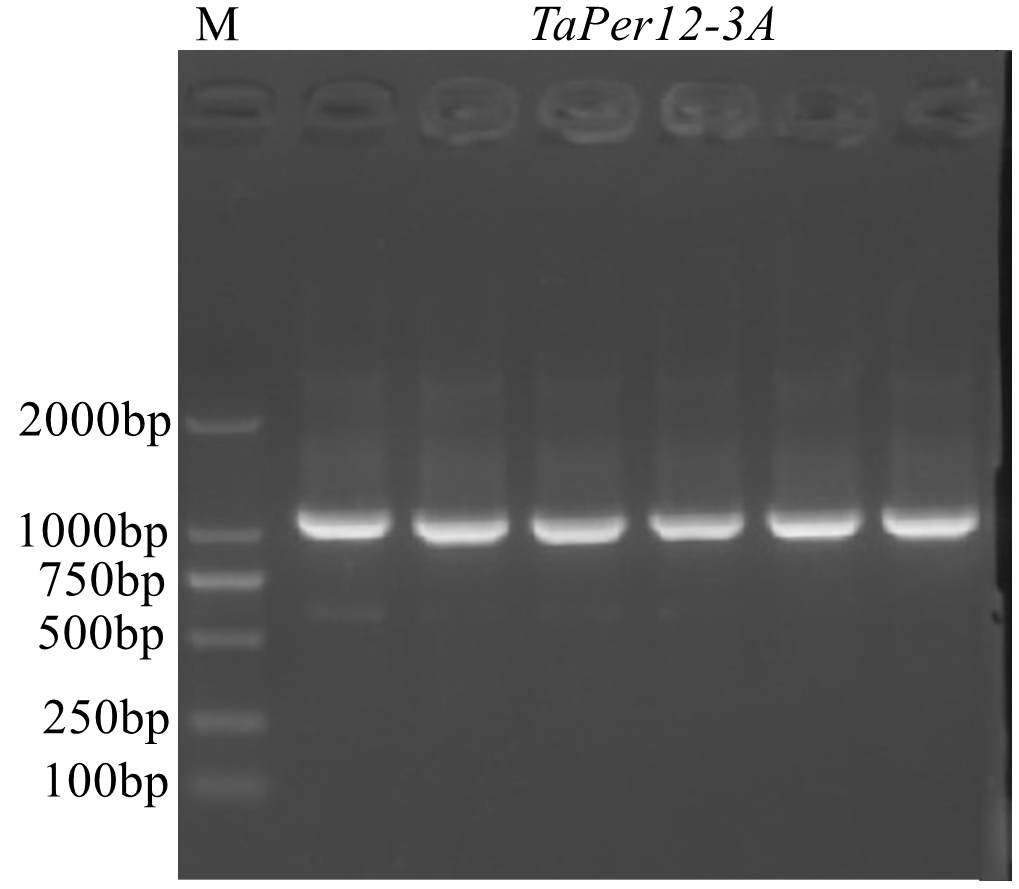


**Fig. S5. The gel image of the *TaPer12-3A* gene detected in wheat variety WTB**


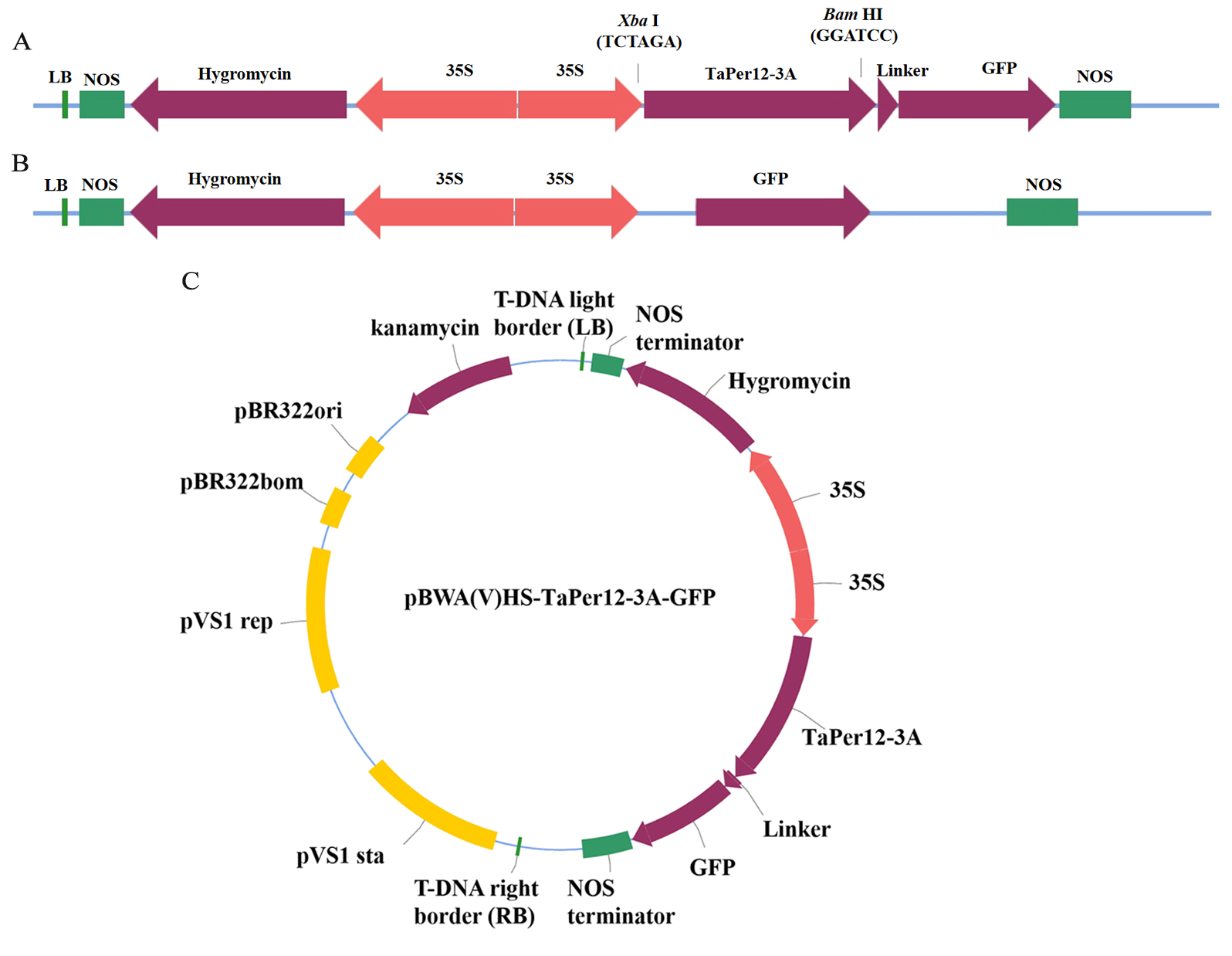


**Fig. S6. Map of TaPer12-3A subcellular localization vector**

**A**. Linear map of TaPer12-3A subcellular localization vector. **B**. Subcellular localization control vector. C. Loop map of TaPer12-3A subcellular localization vector.


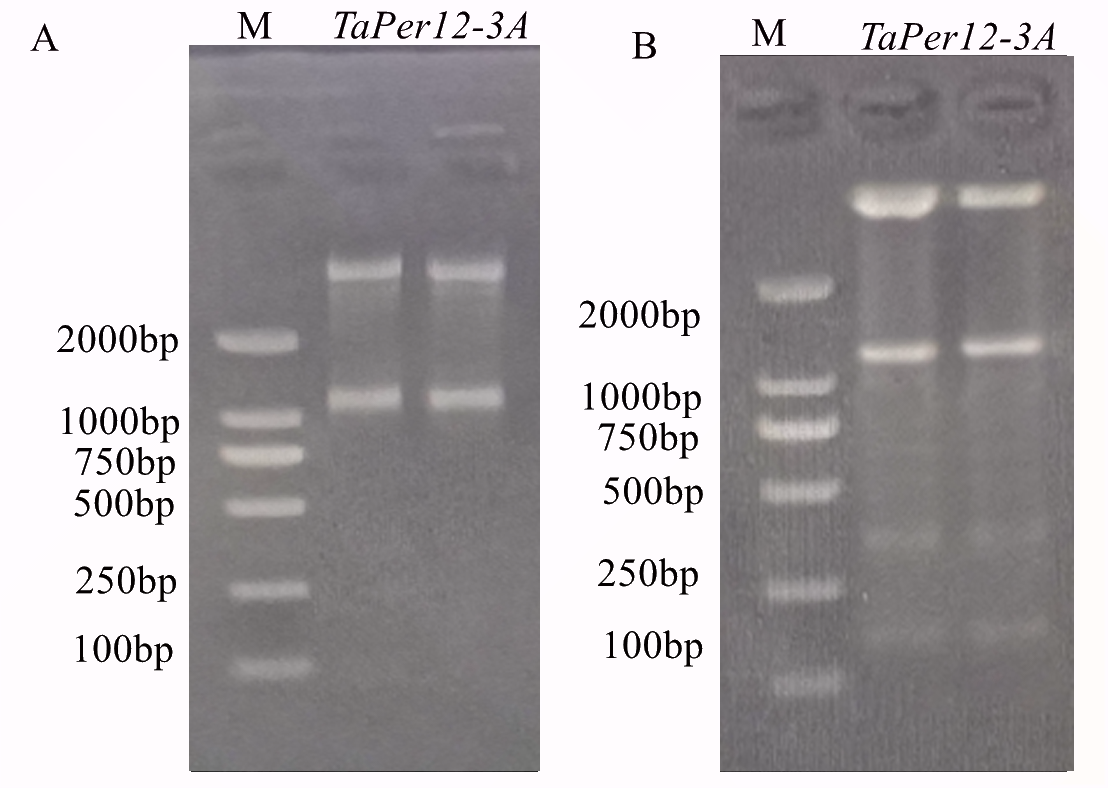


**Fig. S7. Validation of the *TaPer12-3A* expression vector by double enzyme digestion**

**A**. Validation of the *TaPer12-3A* overexpression expression vector by double enzyme digestion. **B**. Validation of *TaPer12-3A* subcellular localization expression vector by double enzyme digestion.


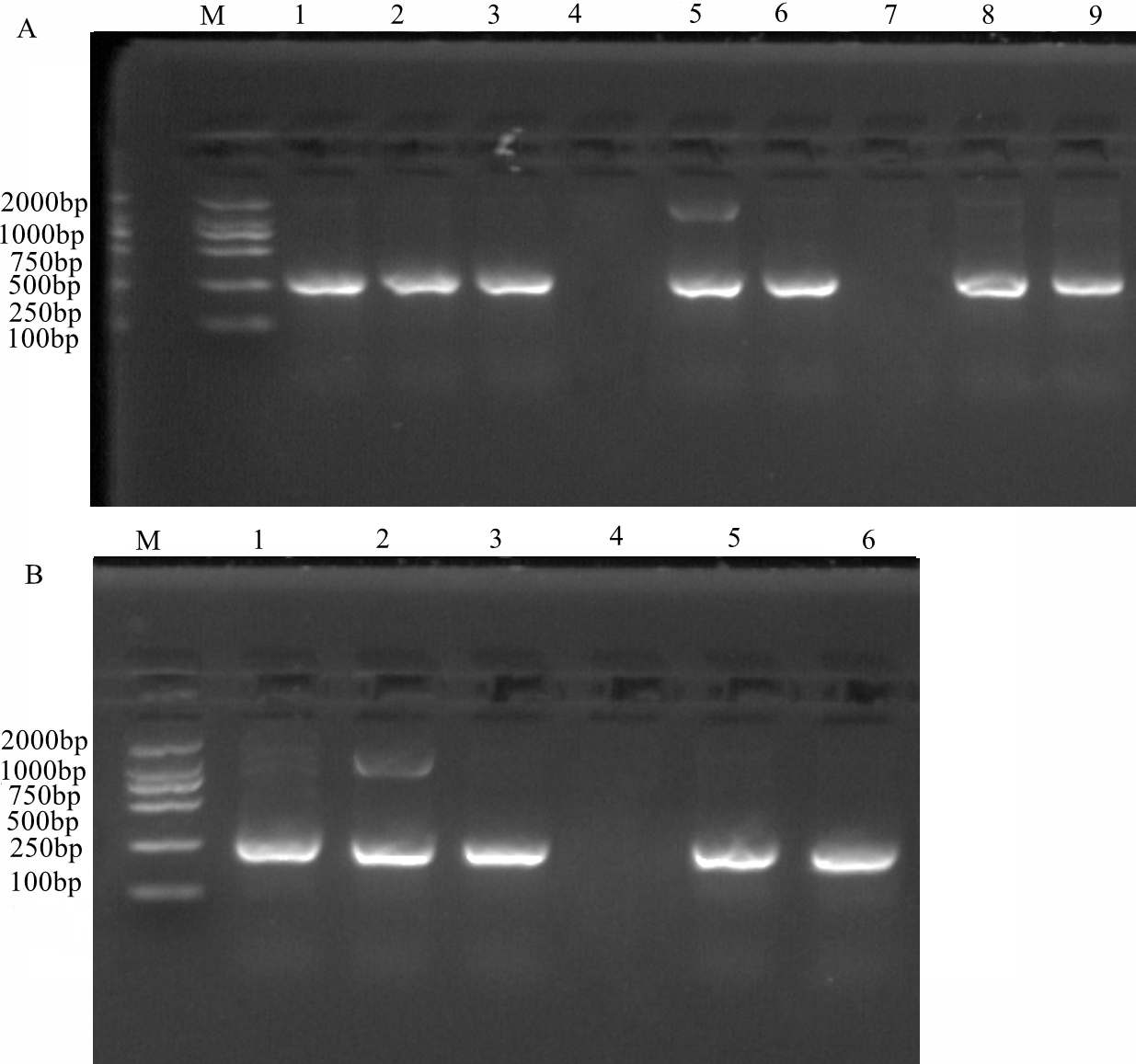


**Fig. S8. Detection of the *TaPer12-3A* overexpressing *Arabidopsis* and rice positive plants**

**A**. Detection of the *TaPer12-3A* overexpression *Arabidopsis* positive plants. **B**. Detection of the *TaPer12-3A* overexpression rice positive plants.


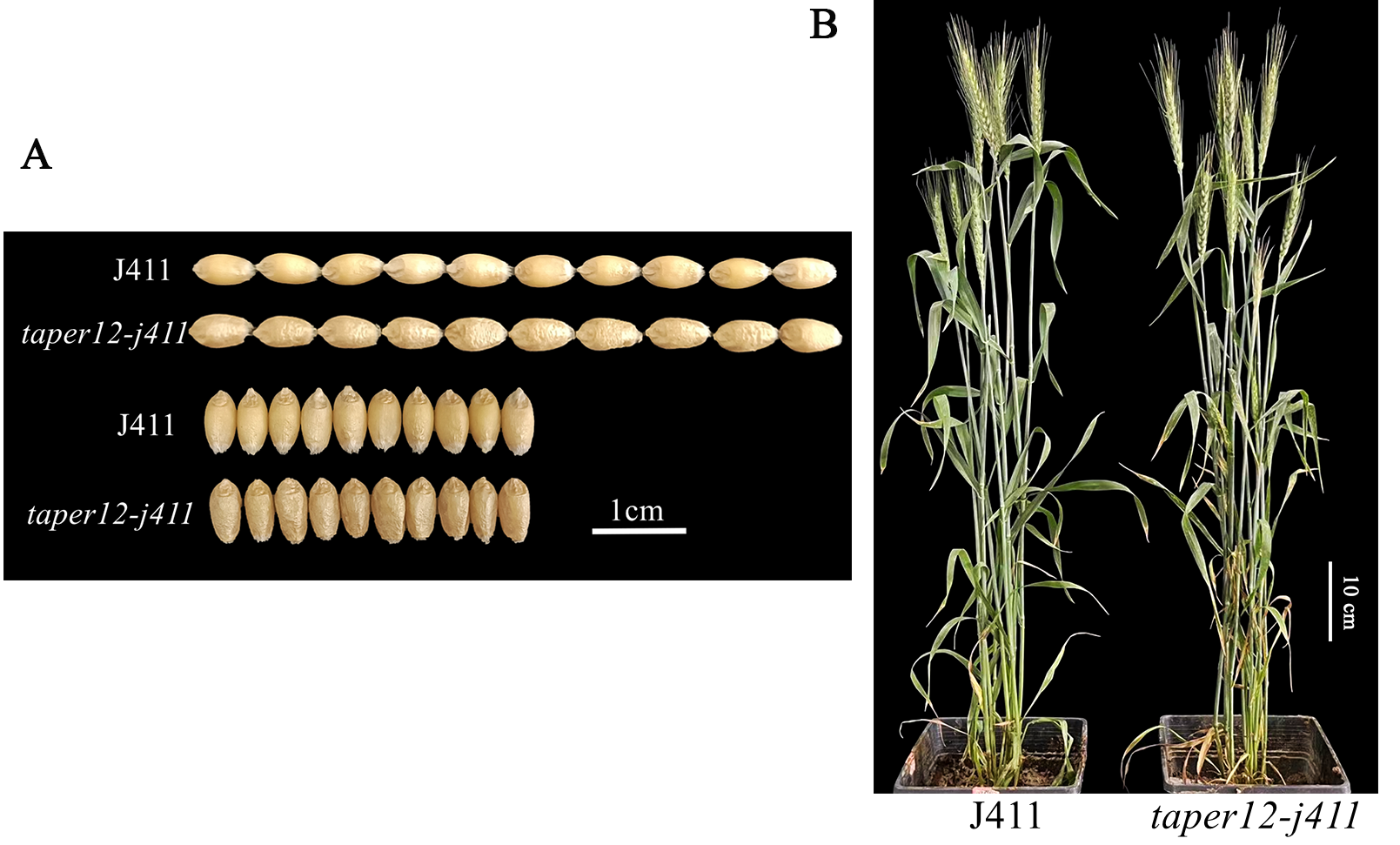


**Fig. S9. Investigation of agronomic characteristics of wheat variety J411 (wild type) and the EMS mutant *taper12-j411***

**A**. Images of J411 and *taper12-j411* seeds. **B**. Images of J411 and *taper12-j411* plants.

**Table S1. Specific primers used in this study.**

| **Experiments** | **name** |  | **Primer sequence (5′→3′)** | **TM** |
| --- | --- | --- | --- | --- |
| qRT-PCR | *TaActin* | Forward | CCTCTCTGCGCCAATCGT | 60℃ |
|  |  | Reverse | TCAGCCGAGCGGGAAATTGT | 60℃ |
|  | *OsActin* | Forward | AGGAAGGCTGGAAGAGGACC | 60℃ |
|  |  | Reverse | CGGGAAATTGTGAGGGACAT | 60℃ |
|  | *AtActin* | Forward | GTATGTGGCTATTCAGGCTGT | 60℃ |
|  |  | Reverse | CTGGCGGTGCTTCTTCTCTG | 60℃ |
|  | *TaGA20ox2 TraesCS3B02G439900* | Forward | CAACGTCGGCGACATCAT | 60℃ |
|  |  | Reverse | GAGTTCACCGACACCCTG | 60℃ |
|  | *TaGA2ox1 TraesCS1B02G123500* | Forward | AGAAAGCAGGAAACCAAACA | 60℃ |
|  |  | Reverse | TCAGACAGCAGGAGAGTG | 60℃ |
|  | *TaNCED2 TraesCS5A02G374000* | Forward | GGAGATGGAAAGAGGAAGTCG | 60℃ |
|  |  | Reverse | GAAGCAAGTGTGAGCTAAC | 60℃ |
|  | *TaABA8'OH2 TraesCS5D02G244900* | Forward | ACGTCGAGTACAGCCCATTC | 60℃ |
|  |  | Reverse | TCGTCGTCGTCGTAGTTGTC | 60℃ |
| gene cloning | *TaPer12-3A* | TaPerF | AAGGTCACCTACACGTACCTAGC | 57℃ |
|  |  | TaPerR | CAAGGAAACATAAGCATCGATC | 57℃ |
| expression primers | *35S:TaPer12* | Forward | TGCGAATCCGCAAAGCAGTAGAGAGC | 72℃ |
|  |  | Reverse | CGCGGATCCTTGAGGCTGGGTGTAATC | 72℃ |
| localization primers | *TaPer12:GFP* | Forward | TGCTCTAGAGCAAAGCAGTAGAGAGC | 72℃ |
|  |  | Reverse | CGCGGATCCTTGAGGCTGGGTGTAATC | 72℃ |
| gene sequencing | M13 | Forward | ACTGGCCGTCGTTTTAC | 60℃ |
|  |  | Reverse | GTCATAGCTGTTTCCTG | 60℃ |
| overexpression detection | Detect the  transgenic plants | Forward | CGGCGCAAGACGTTCTC | 60℃ |
|  |  | Reverse | GTGGTGGCGTTGGTGAAG | 60℃ |

**Table S2.** Expression data of *TaPer12-3A* in seeds of Waitoubai (WTB) and other wheat tissues.

| RNA-seq | | 28DPA-NT | 28DPA-HT | 35DPA-NT | 35DPA-HT |
| --- | --- | --- | --- | --- | --- |
|  |  | 4.854787879 | 9.337904762 | 3.43878788 | 11.14718615 |
|  |  | 3.587454545 | 9.667974026 | 3.78225974 | 12.14718615 |
|  |  | 4.362424242 | 10.16761905 | 3.120865801 | 13.06618182 |
| qRT-PCR | | 28DPA-NT | 28DPA-HT | 35DPA-NT | 35DPA-HT |
|  |  | 6.32833 | 7.413028 | 5.064952 | 9.539404 |
|  |  | 5.77714 | 11.897242 | 5.746827 | 12.743986 |
|  |  | 6.471551 | 12.037075 | 4.766283 | 12.732601 |
| RNA-seq | root | stem | leaf | spike | grain |
|  | 0 | 0.009855 | 0.002022 | 0.019561 | 0.748813 |
|  | 0 | 0.007215 | 0.001481 | 0.004967 | 0.642593 |
|  | 0 | 0.026925 | 0.005525 | 0.034155 | 0.855033 |
| qRT-PCR | root | stem | leaf | spike | grain |
|  | 0 | 0.02542848 | 0.00538446 | 0.0351912 | 1.526 |
|  | 0 | 0.0186147 | 0.00394482 | 0.050680313 | 1.616267 |
|  | 0 | 0.06947166 | 0.00471374 | 0.0103824 | 1.7737 |

**Table S3.** Expression data of *TaPer12-3A* in overexpression *Arabidopsis* and rice lines and germination phenotypes of transgenic seeds.

| qRT-PCR | Col-0 | ***At-L2*** | ***At-L6*** | ***At-L9*** | Nip | ***35S:TaPer12-2*** | ***35S:TaPer12-3*** | ***35S:TaPer12-5*** |
| --- | --- | --- | --- | --- | --- | --- | --- | --- |
|  | 0.00 | 12.36 | 16.17 | 16.12 | 0.00 | 6.62 | 4.77 | 5.47 |
|  | 0.00 | 15.08 | 17.12 | 18.84 | 0.00 | 5.74 | 3.48 | 5.04 |
|  | 0.00 | 14.73 | 14.34 | 16.51 | 0.00 | 6.50 | 4.79 | 4.95 |
| GP (%) | Col-0 | ***At-L2*** | ***At-L6*** | ***At-L9*** | Nip | ***35S:TaPer12-2*** | ***35S:TaPer12-3*** | ***35S:TaPer12-5*** |
|  | 82 | 93 | 98 | 97 | 71 | 98 | 88 | 96 |
|  | 86 | 98 | 96 | 100 | 74 | 96 | 91 | 93 |
|  | 79 | 95 | 99 | 100 | 74 | 94 | 95 | 90 |

**Table S4.** Gibberellic acid (GA), abscisic acid (ABA), and hydrogen peroxide (H_2_O_2_) contents and peroxidase (POD) activity data of *taper12-j411* and J411 seeds.

| GA (pmol/ml) | J411 | | | *taper12-j411* | | |
| --- | --- | --- | --- | --- | --- | --- |
| 0 h | 30.79 | 26.86 | 29.21 | 23.81 | 20.23 | 17.72 |
| 24 h | 40.49 | 35.65 | 38.00 | 19.20 | 23.38 | 21.27 |
| 48 h | 42.32 | 38.89 | 42.18 | 23.38 | 21.55 | 22.58 |
| 72 h | 42.28 | 42.63 | 37.93 | 31.98 | 29.21 | 33.91 |
| ABA (pmol/ml) | J411 | | | *taper12-j411* | | |
| 0 h | 18.27 | 19.73 | 21.36 | 30.47 | 20.94 | 27.32 |
| 24 h | 11.63 | 12.10 | 16.44 | 19.96 | 24.03 | 21.17 |
| 48 h | 11.72 | 10.65 | 9.24 | 20.62 | 18.46 | 22.49 |
| 72 h | 7.00 | 7.35 | 5.01 | 17.39 | 20.99 | 16.07 |
| POD (U/g) | J411 | | | *taper12-j411* | | |
| 0 h | 296.61 | 265.45 | 280.04 | 225.65 | 200.20 | 179.32 |
| 24 h | 324.29 | 315.15 | 296.45 | 238.21 | 202.78 | 203.70 |
| 48 h | 400.79 | 405.68 | 374.81 | 225.62 | 213.27 | 230.25 |
| 72 h | 404.69 | 376.91 | 447.59 | 230.05 | 300.88 | 279.10 |
| H_2_O_2_ (U/g) | J411 | | | *taper12-j411* | | |
| 0 h | 18.07 | 15.95 | 18.44 | 16.22 | 22.15 | 19.01 |
| 24 h | 20.32 | 23.63 | 27.94 | 31.22 | 34.67 | 30.77 |
| 48 h | 22.34 | 25.03 | 30.53 | 38.41 | 44.86 | 40.95 |
| 72 h | 29.19 | 24.63 | 27.81 | 48.30 | 45.20 | 42.44 |

**Table S5.** Expression data of the key genes involved in GA and ABA biosynthesis and catabolism pathways in *taper12-j411* and J411 seeds.

| *TaGA20ox2* | J411 | | | *taper12-j411* | | |
| --- | --- | --- | --- | --- | --- | --- |
| 0 h | 4.81 | 3.49 | 3.78 | 2.21 | 2.13 | 2.80 |
| 24 h | 6.55 | 6.12 | 4.69 | 3.89 | 2.32 | 2.82 |
| 48 h | 7.47 | 6.27 | 6.31 | 4.92 | 4.09 | 4.36 |
| 72 h | 9.25 | 8.17 | 9.44 | 5.22 | 6.13 | 6.77 |
| *TaGA2ox1* | J411 | | | *taper12-j411* | | |
| 0 h | 6.88 | 5.46 | 6.39 | 8.92 | 6.98 | 8.64 |
| 24 h | 5.30 | 5.14 | 3.78 | 6.14 | 7.32 | 6.05 |
| 48 h | 3.07 | 3.48 | 3.26 | 5.09 | 4.90 | 5.74 |
| 72 h | 1.39 | 1.62 | 1.63 | 2.09 | 3.10 | 2.69 |
| *TaNCED2* | J411 | | | *taper12-j411* | | |
| 0 h | 8.16 | 11.23 | 9.44 | 18.45 | 20.28 | 21.45 |
| 24 h | 5.59 | 7.44 | 7.47 | 13.63 | 15.14 | 10.57 |
| 48 h | 4.05 | 3.18 | 3.84 | 8.98 | 12.04 | 8.06 |
| 72 h | 1.93 | 2.10 | 3.11 | 6.09 | 8.25 | 5.92 |
| *TaABA8'OH2* | J411 | | | *taper12-j411* | | |
| 0 h | 1.86 | 2.80 | 2.80 | 1.59 | 1.53 | 1.16 |
| 24 h | 4.61 | 5.61 | 5.13 | 2.66 | 2.15 | 2.43 |
| 48 h | 6.40 | 5.92 | 6.45 | 2.60 | 3.36 | 2.56 |
| 72 h | 5.47 | 6.72 | 5.86 | 3.55 | 2.82 | 2.60 |
